# Strong impact of natural-selection–free heterogeneity in genetics of age-related phenotypes

**DOI:** 10.18632/aging.101407

**Published:** 2018-03-29

**Authors:** Alexander M. Kulminski, Jian Huang, Yury Loika, Konstantin G. Arbeev, Olivia Bagley, Arseniy Yashkin, Matt Duan, Irina Culminskaya

**Affiliations:** 1 Biodemography of Aging Research Unit, Social Science Research Institute, Duke University, Durham, NC 27708, USA

**Keywords:** genome-wide association studies, pleiotropy, age-related phenotypes, antagonistic genetic heterogeneity, aging, health span, life span

## Abstract

A conceptual difficulty in genetics of age-related phenotypes that make individuals vulnerable to disease in post-reproductive life is genetic heterogeneity attributed to an undefined role of evolution in establishing their molecular mechanisms. Here, we performed univariate and pleiotropic genome-wide meta-analyses of 20 age-related phenotypes leveraging longitudinal information in a sample of 33,431 individuals and dealing with the natural-selection–free genetic heterogeneity. We identified 142 non-proxy single nucleotide polymorphisms (SNPs) with phenotype-specific (18 SNPs) and pleiotropic (124 SNPs) associations at genome-wide level. Univariate meta-analysis identified two novel (11.1%) and replicated 16 SNPs whereas pleiotropic meta-analysis identified 115 novel (92.7%) and nine replicated SNPs. Pleiotropic associations for most novel (93.9%) and all replicated SNPs were strongly impacted by the natural-selection–free genetic heterogeneity in its unconventional form of antagonistic heterogeneity, implying antagonistic directions of genetic effects for directly correlated phenotypes. Our results show that the common genome-wide approach is well adapted to handle homogeneous univariate associations within Mendelian framework whereas most associations with age-related phenotypes are more complex and well beyond that framework. Dissecting the natural-selection–free genetic heterogeneity is critical for gaining insights into genetics of age-related phenotypes and has substantial and unexplored yet potential for improving efficiency of genome-wide analysis.

## Introduction

Genome-wide association studies (GWAS) are a powerful tool for hypothesis-free analysis of genetic predisposition to various phenotypes. Historically, GWAS were built within the “common disease – common genetic variant” concept following the framework of medical genetics. The underlying hypothesis in this framework is that there is a “true” or causal genetic effect on a phenotype of interest [1,2]. This approach in GWAS has been supported by successful discovery of causal genetic mutations for Mendelian disorders [3]. Essentially the same approach is pursued in GWAS of complex phenotypes, which do not follow clear pattern of Mendelian inheritance [4]. Extension of the framework of medical genetics of hereditary disorders to the complex non-Mendelian phenotypes relies on the hypothesis that they can have a genetic component. This hypothesis is supported by the concept of heritability, with significant heritability interpreted as indication of “pure” or “true” genetic component in a trait [2]. The concept of heritability, introduced in breeding experiments to improve crop yield, requires, however, controlled and fixed environment [5,6] that is strongly violated in human populations.

The problem becomes even more challenging for age-related phenotypes, i.e., phenotypes that make individuals vulnerable to disease in post-reproductive life. A conceptual difficulty in genetics of age-related phenotypes is the natural-selection–free genetic heterogeneity attributed to an undefined role of evolution in establishing their molecular mechanisms [7,8]. This problem is complicated by recent changes in human life expectancy [9] and the fitness landscape [10-13]. Accordingly, in evolutionary biology, age-related phenotypes are viewed as the results of *indirect* mechanisms (“side effects”) such as co-evolution with fast-evolving pathogens, mismatch with environments, reproductive success at the expense of health, trade-offs that leave every trait suboptimal, defenses and their special costs, etc [7]. The concept of heritability in the evolutionary framework for age-related phenotypes becomes even more problematic because the estimates of heritability change as environment changes [5,6] and significant heritability does not imply that the same genetic variant carry the same risk in different population groups, even of the same ancestry [11].

The natural-selection–free genetic heterogeneity has not been addressed in mainstream GWAS. This heterogeneity implies that differences in genetic predisposition to age-related phenotypes across different population groups is biologically motivated. Accordingly, different, even antagonistic, effects of the same allele on the same phenotype in different population groups are biologically plausible [14,15]. Another challenge in the evolutionary framework is that genetic variants predisposing to a phenotype may not necessarily predispose to another, even causally related, phenotype [16,17] or such genetic variants can predispose to seemingly unrelated phenotypes [18-20].

Here, we examine genetic predisposition to age-related phenotypes following the concept of an undefined role of evolution in establishing their molecular mechanisms. We performed the univariate and pleiotropic GWAS meta-analyses of 20 age-related phenotypes in the sample of 33,431 Caucasians from five longitudinal studies. We identified 142 non-proxy (defined as linkage disequilibrium [LD] *r^2^*<70%) single nucleotide polymorphisms (SNPs) with genome-wide (GW) significance (*p*<*p_GW_*=5×10^-8^). They include two novel and 16 previously reported SNPs attained GW significance in univariate meta-analysis of individual phenotypes, and 115 novel and nine previously reported SNPs attained GW significance in pleiotropic meta-analysis. We show that pleiotropic associations for most novel SNPs, 93.9% (108 of 115 SNPs), and for all replicated SNPs were strongly affected by the natural-selection–free genetic heterogeneity in a rarely recognized form of antagonistic heterogeneity, implying antagonistic directions of genetic effects for directly correlated phenotypes.

## RESULTS

### Study overview

**Figure 1:**
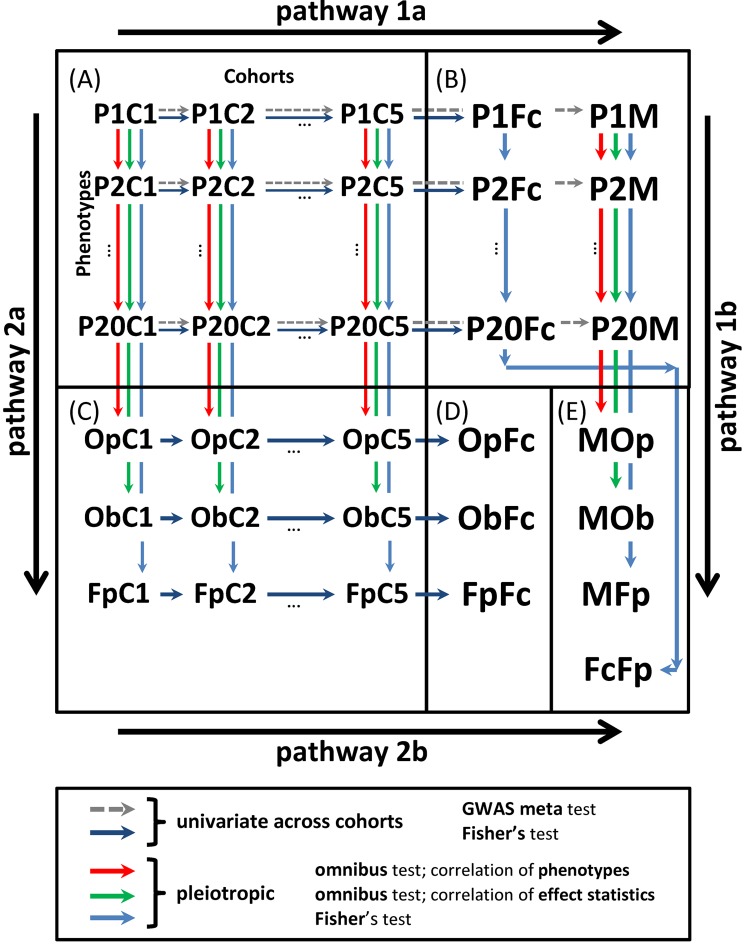
**Scheme of univariate and pleiotropic meta-analyses in stage 2**. (**A**) Statistics from stage 1 univariate GWAS of 20 phenotypes in five cohorts denoted P*i*C*j*, $i\in \left(\overset{-}{1,20}\right)$ and $j\in \left(\overset{-}{1,5}\right)$. (**B**) Univariate statistics from the meta-analysis across cohorts using the fixed-effects meta-test (P*i*M) and Fisher test (P*i*Fc). (**C**) Statistics from the pleiotropic meta-analysis across phenotypes in cohort *j* for: (i) omnibus tests with correlation matrix for phenotypes $\Sigma_{j}^{P}$ (OpC*j*) and effect statistics $\Sigma_{j}^{B}$ (ObC*j*) and (ii) Fisher test (FpC*j*). (**D**) Meta-statistics from Fisher test across cohorts for the results in (**C**) (OpFc, ObFc, and FpFc). (**E**) Meta-statistics across phenotypes for the results in (**B**) from (i) meta-test across cohorts and omnibus test across phenotypes with correlation matrix for phenotypes $\Sigma_{m}^{P}$ (MOp), (ii) meta-test across cohorts and omnibus test across phenotypes with correlation matrix for effect statistics $\Sigma_{m}^{B}$ (MOb), (iii) meta-test across cohorts and Fisher test across phenotypes (MFp), and (iv) Fisher test across cohorts and Fisher test across phenotypes (FcFp). Pathway 1: meta-analysis combining statistics across cohorts (pathway 1a) and pleiotropic meta-analysis across phenotypes (pathway 1b). Pathway 2: meta-analysis combining statistics across phenotypes (pathway 2a) and cohorts (pathway 2b).

We performed two-stage univariate and pleiotropic meta-analyses of genetic predisposition to 20 weakly-to-modestly correlated age-related phenotypes (Supplementary Figure 1). The data were drawn from five longitudinal studies for 33,431 men and women combined, who identified themselves as of Caucasian ancestry. In stage 1, we first performed simplified univariate GWAS of each phenotype in each cohort separately using *plink* software [21]. Then, these results were prioritized and top 1,000 promising SNPs were selected for more comprehensive analysis leveraging information on repeated measurements for quantitative markers and timing of the risk outcomes (Tables 1 and Supplementary Table 1). In stage 2, we performed univariate meta-analysis to combine statistics for these 1,000 selected SNPs across cohorts and pleiotropic meta-analysis to combine such statistics across phenotypes. These analyses addressed the natural-selection–free genetic heterogeneity in genetic predisposition to age-related phenotypes (see the Introduction) by performing five meta-tests (Fig. 1) (details in Methods).

**Table 1:** Basic characteristics of cohorts included in the analyses.

**Cohort**	**Sample**	**Number**	**Age (SD),**	**Birth dates**	**Quantitative markers**	**Risk outcomes**
	**size**	**of visits**	**years**	**(range)**		
**Markers:** BG, BMI, CRP, creatinine, DBP, FVC, HC, HDL-C, HR, SBP, TC, TG						
**Risk outcomes:** AD, AF, cancer, CHD, DM, death, HF, stroke						
ARIC	9,612	4	54.3 (5.7)	1921-1944	All	All except AD
CHS	3,182	10	72.4 (5.4)	1885-1925	All	All
FHS	8,628	28*	37.8 (9.3)	1885-1980	All	All
MESA	2,527	5	64.3 (10.2)	1917-1957	All	All except cancer and AD
HRS	9,482	2	58.2 (9.1)	1905-1974	All except creatinine, FVC, HC, HR, and TG	All except AF

**Cohort**: Atherosclerosis Risk in Communities Study (ARIC); Cardiovascular Health Study (CHS); Framingham Heart Study (FHS), the Multi-Ethnic Study of Atherosclerosis (MESA), and Health and Retirement Study (HRS).

**Age** is given at baseline; standard deviation (SD).

*Number of visits in the FHS original cohort, offspring and 3^rd^ generation cohort are 28, 8, and 2, respectively.

**Quantitative markers**: blood glucose (BG); body mass index (BMI); C-reactive protein (CRP); creatinine; diastolic blood pressure (DBP); forced vital capacity (FVC); heart rate (HR); hematocrit (HC); high-density lipoprotein cholesterol (HDL-C); systolic blood pressure (SBP); total cholesterol (TC); and triglycerides (TG).

**Risk outcomes**: Alzheimer’s disease and related dementias (AD), atrial fibrillation (AF); cancer; coronary heart disease (CHD); diabetes mellitus (DM); death; heart failure (HF); and stroke.

Additional details are provided in Supplementary Table 1.

### Univariate meta-analysis

Using the Fisher test and a fixed-effect meta-test (Fig. 1, pathway 1a), we identified two novel (see Methods) non-proxy SNPs (defined as LD *r^2^*<70%, actual LD for the reported SNPs in the same locus ranged *r^2^*=1% to 64%, Supplementary Table 2) associated at GW level with HC (rs6745983, *β_meta_*=-0.077, *p_meta_*=4.2×10^-8^) and BG (rs10885409, *β_meta_*=0.672, *p_meta_*=1.9×10^-8^) (Supplementary Table 3). We also replicated (*p*<*p_GW_*) 24 associations, primarily with lipids (21 associations), for 16 SNPs in or nearby (within ±100 Kb flanking region for the index SNP) 11 protein coding genes (Supplementary Table 3). Seven associations were replicated in the *LPL*-gene locus. *P-*values were smaller for the meta-test than for the Fisher test (*p_meta_*<*p_Fisher_*) for all associations except for the associations of rs780094 with BG (*p_meta_*=2.7×10^-8^ vs. *p_Fisher_* =2.4×10^-11^) and rs261332 with TC (*p_meta_*=5.1×10^-8^ vs. *p_Fisher_* =2.7×10^-9^). The Fisher test benefited the meta-analysis because it disregarded strong heterogeneity in the effect sizes across cohorts measured by the heterogeneity coefficient *I^2^*, i.e., *I^2^*=87.8% for rs780094 and *I^2^*=82.2% for rs261332, as supported by significant inverse correlation of the ratio log_10_(*p_meta_*)/log_10_(*p_Fisher_*) with *I^2^*, *r_Pearson_*=-0.634, *p*=5.0×10^-4^.

Significance of the association of rs2155216 with TG in our modest sample of 33,431 individuals (*p_meta_*=9.4×10^-14^) was about the same as previously reported (*p_grasp_*=1.6×10^-14^) in a sample of 140,059 individuals [22]. These *p*-values indicate improvement of the efficiency of the analysis that can be quantified by the ratio of the log_10_(*p*-value) to the sample size [23] yielding 4-fold (=log_10_(9.4×10^-14^)×140,059/log_10_(1.6×10^-14^)/33,431) larger efficiency in our study than in [22]. This improvement was partly achieved by leveraging longitudinal information on repeated measurements that resulted in smaller standard errors (Supplementary Figure 2).

### Pleiotropic meta-analysis

We used four tests in pathway 1 and three tests in pathway 2 (Fig. 1) to examine pleiotropy in the domains of 12 quantitative markers, 8 diseases and death, and all 20 phenotypes (Table 1). Pleiotropic associations were defined as at least one test showing GW significance (see Methods).

This analysis identified 115 novel pleiotropic SNPs in or nearby (within ±100 Kb flanking region for the index SNP) 84 protein coding genes (Table 2) and replicated nine SNPs (Supplementary Table 4) with *p_pleio_*<*p_GW_* by combining associations with multiple phenotypes that individually did not attain GW significance in univariate meta-analysis (*p_uni_*>*p_GW_*) (Supplementary Table 5). SNPs were identified as novel if they did not attain GW significance in our pleiotropic meta-analysis of the results collected in GRASP for the index SNPs or for flanking SNPs, if no results for 20 selected phenotypes (Figure 1) were reported in GRASP for the index SNPs (see Methods). Specifically, of 115 SNPs, 31 were not reported in GRASP for the selected 20 phenotypes. For them, we identified multiple SNPs within ±1Mb flanking region in GRASP. For the flanking SNPs, which were in the strongest LD to the index SNPs, no GW significant pleiotropic associations were identified (Table 3). Nine flanking SNPs showed GW significant pleiotropic effects, which were independent of the pleiotropic effects for the index SNPs. Of 115 novel SNPs, 29 SNPs attained smaller *p*-value in pathway 1 and the remaining 86 SNPs in pathway 2.

**Table 2:** Novel SNPs attained genome-wide significance in pleiotropic meta-analysis.

**ID**	**Gene(s)^1^**	**SNP^2^**	**Chr**	**Location,** **base pairs** **GRCh38**	**EA**	**EAF**	**Domain**	**N_P_**	**Pathway**	**Meta *p*-values**				**Group**	***P*_GRASP_**	**N_G_**
									**1**	**P_MOp_**	**P_MOb_**	**P_MFp_**	**P_FcFp_**			
									**2**	**P_OpFc_**	**P_ObFc_**		**P_FpFc_**			
1	CLCN6	rs17376328	1	11,816,605	A	0.051	12M	3	2	6.89E-15	1.21E-05		2.83E-07	HP	3.08E-07	3
2	NKAIN1-SNRNP40/ ZCCHC17*	rs7549339	1	31,249,319	A	0.218	12M	5	2	4.89E-10	3.69E-15		2.74E-06	HP/HB	3.33E-01	1
3	TINAGL1-SERINC2*	rs16834550	1	31,530,916	A	0.125	12M	8	2	4.17E-10	5.48E-09		1.04E-08	M		0
4	CSMD2	rs10914845	1	34,022,514	C	0.499	20P	3	1	2.23E-08	1.73E-03	1.41E-03	3.97E-03	HP	3.20E-01	1
5		rs7551194	1	38,680,229	A	0.249	12M	4	1	2.55E-08	3.99E-02	2.16E-04	3.92E-02	HP	1.66E-01	2
6	AKIRIN1/ NDUFS5-MACF1*	rs7554809	1	39,038,206	A	0.168	12M	3	2	2.55E-08	4.16E-05		1.30E-03	HP	6.04E-02	2
7		rs778405	1	56,237,741	A	0.493	12M	2	2	1.38E-11	2.34E-05		4.20E-06	HP	2.98E-01	1
8	PTGER3	rs1327464	1	70,900,555	A	0.339	12M	5	1	2.37E-16	1.17E-03	7.39E-07	2.04E-05	HP	3.14E-01	1
9		rs17105569	1	80,872,915	A	0.136	12M	4	1	1.80E-09	6.06E-03	2.96E-04	1.36E-02	HP	1.62E-01	1
10	RNPC3/ AMY2B-AMY2A*	rs4847151	1	103,581,040	T	0.365	20P	5	2	1.04E-09	5.48E-04		1.45E-02	HP	3.23E-01	1
11	NTNG1	rs7542677	1	107,319,431	T	0.1	12M	5	2	2.99E-09	1.94E-06		6.08E-04	HP	1.03E-01	2
12	FCRL4-FCRL3*	rs1969742	1	157,643,547	C	0.317	12M	4	2	1.58E-12	1.60E-08		4.71E-05	HP/HB		0
13	OLFML2B	rs2490420	1	161,994,746	G	0.31	20P	5	2	2.49E-08	1.02E-02		2.92E-03	HP	3.15E-01	1
14	PRRX1	rs9426908	1	170,674,846	C	0.069	20P	7	2	5.97E-07	7.58E-10		2.24E-05	HB	3.09E-01	1
15	KCNH1	rs1934628	1	210,739,125	G	0.164	12M	5	2	8.52E-10	1.98E-07		1.78E-05	HP		0
16	ESRRG	rs1436879	1	216,909,043	A	0.041	20P	5	2	1.68E-07	8.21E-09		6.72E-07	M	2.91E-01	1
17	GREB1	rs7596162	2	11,604,106	C	0.196	12M	5	1	3.83E-08	2.03E-04	3.02E-06	9.25E-04	M	3.18E-01	1
18	SDC1-LAPTM4A*	rs11685359	2	20,149,672	T	0.137	12M	3	1	2.80E-09	4.04E-03	6.86E-06	7.49E-04	HP	3.15E-01	1
19	ADCY3-DNAJC27*	rs10182181	2	24,927,427	C	0.472	12M	3	2	8.48E-10	3.73E-06		4.44E-05	HP	3.41E-05	3
20	CAPN13	rs10173959	2	30,806,079	A	0.297	20P	6	2	8.25E-11	5.43E-04		4.89E-05	HP	1.66E-02	3
21	CAPN13	rs10176484	2	30,806,230	A	0.304	20P	4	2	6.03E-10	1.49E-04		6.44E-05	HP	1.88E-02	3
22		rs10495824	2	34,525,478	C	0.247	12M	3	2	3.61E-17	4.09E-11		1.03E-03	HP/HB	1.75E-01	1
23	THADA	rs17334919	2	43,480,246	A	0.1	12M	4	2	2.89E-14	3.96E-10		5.19E-07	HP/HB	1.17E-04	3
24	EPAS1	rs10191091	2	46,346,071	G	0.498	12M	7	2	1.48E-08	2.13E-11		5.63E-08	HB	1.43E-01	2
25	ANTXR1	rs4255990	2	69,046,532	C	0.062	20P	7	2	1.02E-11	1.67E-07		7.89E-10	M	8.35E-02	1
26	DNAH6	rs11889456	2	84,619,559	C	0.077	20P	7	1	8.17E-12	7.78E-05	1.16E-07	1.11E-04	HP	1.65E-01	1
27	MGAT5	rs755503	2	134,386,883	A	0.427	12M	4	2	5.89E-11	2.13E-04		1.75E-04	HP		0
28	TMEM163	rs503562	2	134,502,500	G	0.474	12M	5	2	9.26E-21	1.37E-14		7.52E-06	HP/HB	2.62E-02	2
29	TMEM163	rs666614	2	134,532,882	G	0.416	12M	5	1	1.50E-09	3.13E-07	1.93E-07	3.96E-09	HP	2.66E-03	4
30	UBXN4	rs6430585	2	135,749,357	A	0.253	12M	5	2	6.09E-11	4.00E-06		3.93E-04	HP	1.69E-04	3
31	CCDC141	rs12693171	2	178,883,720	A	0.132	12M	7	2	7.25E-15	7.51E-09		1.43E-08	HP	1.31E-01	1
32		rs6781156	3	5,811,612	T	0.394	20P	7	1	2.48E-10	3.24E-03	3.97E-05	2.06E-03	HP	6.82E-02	2
33	CCK*	rs11129950	3	42,273,095	C	0.15	12M	5	2	1.10E-10	4.39E-07		1.43E-06	HP	5.44E-02	2
34	WNT5A/ERC2*	rs751194	3	55,453,400	T	0.46	20P	1	2	2.92E-08	1.45E-04		2.80E-02	HP	4.47E-02	2
35	FHIT	rs1716721	3	60,696,259	A	0.126	20P	7	2	1.13E-09	8.92E-06		2.45E-07	HP	3.79E-01	1
36	COL6A5	rs16827675	3	130,428,488	C	0.027	20P	8	2	1.09E-10	7.13E-12		9.13E-10	HB	4.14E-01	1
37	GFM1	rs16829273	3	158,664,831	C	0.155	12M	7	2	3.42E-12	2.07E-08		5.50E-06	HP/HB		0
38		rs7676659	4	26,055,991	T	0.414	12M	2	2	4.17E-09	4.21E-07		1.73E-02	HP	5.02E-03	3
39		rs6827919	4	38,389,984	A	0.459	12M	6	1	3.93E-09	4.63E-03	1.01E-04	3.83E-05	HP	4.84E-06	3
40	DCK-SLC4A4*	rs10012631	4	71,093,574	G	0.054	20P	4	2	6.10E-13	5.17E-07		3.03E-05	HP		0
41	SLC4A4	rs6846301	4	71,481,774	G	0.092	20P	9	2	3.44E-12	4.30E-09		4.97E-07	HP/HB	7.95E-02	1
42	CCSER1	rs13103126	4	90,340,833	T	0.448	12M	5	2	2.78E-08	4.49E-06		8.83E-04	HP	9.96E-02	2
43	CCSER1*	rs2176312	4	91,608,453	C	0.478	12M	2	2	6.28E-11	7.48E-07		1.57E-04	HP	1.64E-02	2
44		rs1460770	4	114,484,189	A	0.365	12M	7	2	4.34E-17	1.13E-13		1.09E-08	HP/HB		0
45	USP38-GAB1*	rs300934	4	143,250,595	T	0.334	20P	8	1	1.20E-09	2.82E-03	5.64E-07	1.54E-05	HP	8.15E-03	4
46	FSTL5*	rs7438099	4	161,343,620	T	0.323	12M	8	2	2.24E-08	9.55E-08		6.40E-06	HP	1.20E-01	2
47	CMYA5	rs259130	5	79,763,725	T	0.326	20P	6	2	4.11E-08	5.63E-03		5.65E-04	HP		0
48		rs980831	5	101,415,163	T	0.022	20P	4	1	4.41E-10	3.26E-05	6.18E-04	1.18E-05	HP		0
49	EFNA5	rs152608	5	107,435,508	A	0.247	12M	4	2	5.23E-11	1.12E-11		3.16E-04	HP/HB	1.83E-03	3
50	ADRB2*	rs6580586	5	148,863,160	C	0.116	20P	9	1	8.38E-25	6.29E-09	2.11E-12	9.04E-09	HP	7.17E-03	4
51	GLRA1*	rs10053232	5	152,123,786	T	0.224	12M	3	2	1.19E-09	3.68E-05		2.51E-03	HP	3.43E-01	1
52	EBF1*	rs2434612	5	158,595,033	C	0.207	12M	2	2	3.28E-17	8.25E-11		4.21E-04	HP/HB	6.39E-03	2
53	HLA-E-GNL1/PRR3	rs2844720	6	30,507,940	T	0.312	12M	5	2	3.55E-08	9.56E-05		1.22E-04	HP	8.23E-02	2
54	C6orf10	rs6907322	6	32,357,168	A	0.184	12M	6	1	2.05E-08	6.63E-04	6.60E-06	3.35E-04	HP	2.42E-02	2
55	HLA-DOA-HLA- DPA1/HLA-DPB1	rs6936620	6	33,016,674	T	0.363	20P	7	2	3.18E-09	1.43E-02		5.33E-05	HP		0
56	COL19A1	rs17690160	6	70,197,672	A	0.055	12M	4	1	1.23E-08	5.58E-02	1.13E-02	1.76E-02	HP	5.22E-02	2
57	VGLL2*	rs783199	6	117,229,431	T	0.151	12M	4	2	8.58E-09	1.17E-05		7.04E-04	HP	9.40E-04	4
58		rs9482188	6	121,700,736	A	0.031	12M	5	2	9.97E-17	2.83E-15		4.48E-06	HP/HB		0
59	HDAC9	rs1178348	7	18,176,753	C	0.257	20P	6	2	1.13E-08	1.12E-03		1.70E-04	HP	3.93E-01	1
60	CPVL	rs7785072	7	29,097,886	A	0.497	12M	5	2	3.40E-15	1.34E-09		6.69E-05	HP/HB		0
61	TMEM248*	rs4717331	7	66,913,899	T	0.304	20P	7	2	2.85E-14	7.21E-10		2.68E-08	HP	3.41E-01	1
62	CALCR	rs6966283	7	93,539,572	T	0.108	12M	4	2	5.05E-19	2.92E-10		1.02E-06	HP/HB	2.45E-01	1
63	VGF	rs10953325	7	101,162,227	C	0.401	20P	5	2	5.91E-10	4.37E-07		1.69E-07	HP		0
64		rs7807451	7	115,825,666	A	0.134	20P	8	1	6.20E-09	4.29E-05	3.28E-06	6.43E-06	HP		0
65		rs6466686	7	118,996,763	G	0.356	12M	4	1	1.97E-08	7.48E-03	1.26E-04	8.29E-03	HP		0
66	TSGA13	rs1038638	7	130,676,252	C	0.461	20P	5	1	4.01E-08	9.40E-02	8.55E-03	2.79E-02	HP	1.56E-01	2
67	CNTNAP2	rs700278	7	146,492,560	T	0.458	12M	3	2	4.73E-09	1.26E-05		3.99E-03	HP		0
68	BIN3-EGR3*	rs3893402	8	22,679,688	G	0.321	20P	7	2	8.35E-09	8.09E-04		2.20E-05	HP	4.18E-01	1
69	RP1	rs10103201	8	54,530,554	A	0.377	12M	5	1	1.19E-10	1.66E-04	7.10E-06	1.57E-04	HP	2.27E-01	1
70	XKR9	rs2732091	8	71,006,188	T	0.493	12M	5	1	3.94E-11	9.84E-05	4.10E-06	8.38E-03	HP	1.11E-01	2
71	SLC24A2	rs7851478	9	19,653,532	G	0.294	20P	6	1	7.37E-09	3.01E-03	1.15E-04	2.16E-02	HP		0
72		rs3928808	9	32,278,893	A	0.345	12M	4	2	4.29E-10	1.97E-06		3.48E-03	HP	1.16E-01	2
73	FBP2	rs2987900	9	94,593,188	G	0.057	20P	5	2	1.45E-08	7.07E-02		2.21E-03	HP		0
74	ABCA1	rs4149313	9	104,824,472	C	0.141	20P	5	2	1.39E-09	8.54E-05		2.30E-04	HP		0
75	ABCA1	rs10820738	9	104,828,835	G	0.068	12M	5	2	8.30E-09	2.11E-06		3.34E-03	HP	3.02E-04	2
76	LPAR1	rs1476946	9	111,102,469	C	0.332	12M	5	1	2.47E-08	1.80E-03	3.84E-06	1.26E-02	HP		0
77		rs1340131	10	16,261,811	T	0.028	20P	6	2	2.27E-09	6.96E-04		5.48E-07	HP	3.59E-01	1
78		rs7127823	11	28,939,160	T	0.382	12M	8	2	2.67E-13	1.48E-10		1.66E-07	HP/HB	3.70E-04	4
79	ARL14EP-MPPED2*	rs1222210	11	30,340,578	A	0.201	12M	4	1	1.05E-09	3.88E-02	3.94E-06	3.25E-02	HP	2.68E-01	1
80	SHANK2	rs10459038	11	71,135,915	C	0.485	12M	1	2	1.81E-10	2.42E-04		3.67E-02	HP		0
81	NTM	rs2442100	11	131,429,821	C	0.047	12M	5	2	6.59E-10	3.87E-08		2.29E-05	HP/HB		0
82	SLCO1B7	rs11045743	12	21,089,365	G	0.045	12M	3	2	5.78E-14	2.56E-10		5.23E-07	HP/HB		0
83	FGD4	rs7315682	12	32,533,023	A	0.144	12M	4	2	8.62E-09	1.40E-05		3.86E-03	HP		0
84	RBM19*	rs11066861	12	113,982,465	A	0.057	12M	5	2	3.84E-12	1.20E-08		6.65E-07	HP	1.48E-02	3
85	CDK2AP1	rs1109559	12	123,273,314	C	0.324	20P	4	1	3.10E-09	8.64E-05	1.83E-05	4.01E-03	HP	2.34E-03	4
86		rs9506931	13	22,814,989	G	0.267	12M	5	1	1.05E-08	4.23E-03	3.45E-05	2.00E-02	HP	7.93E-02	2
87	PDX1-CDX2*	rs2504220	13	27,945,217	T	0.114	12M	4	2	4.96E-09	3.83E-05		2.59E-04	HP		0
88	DGKH	rs9315885	13	42,068,674	G	0.365	12M	5	2	5.22E-11	2.56E-09		1.44E-06	HP/HB	3.39E-01	1
89	DGKH	rs670676	13	42,127,603	T	0.208	20P	7	2	1.34E-08	3.03E-06		3.77E-08	M		0
90		rs359412	13	64,653,337	C	0.495	12M	3	2	3.04E-08	4.73E-06		6.27E-02	HP		0
91		rs4903274	14	40,055,761	C	0.296	12M	7	2	4.76E-16	4.43E-09		1.41E-06	HP/HB		0
92	FUT8*	rs4899173	14	65,326,450	T	0.227	20P	7	2	1.90E-08	9.48E-04		2.59E-06	HP		0
93		rs7151718	14	86,428,552	T	0.068	12M	5	2	2.95E-09	8.14E-06		4.84E-04	HP		0
94	RYR3	rs2217807	15	33,491,010	G	0.427	12M	6	1	3.74E-08	1.49E-02	2.59E-04	2.59E-04	HP	1.97E-01	1
95	SHF	rs3959644	15	45,195,934	A	0.35	12M	3	2	4.00E-08	1.03E-05		1.59E-03	HP	3.14E-01	1
96	ATP8B4*	rs626744	15	49,760,008	C	0.026	20P	10	2	1.17E-11	8.33E-10		8.83E-08	HP/HB		0
97	SALL1*	rs7499584	16	51,205,506	A	0.297	20P	4	1	3.27E-12	4.02E-04	2.99E-04	1.85E-04	HP	1.93E-01	2
98	MT3-MT2A*	rs12444489	16	56,597,022	A	0.223	12M	5	2	1.03E-09	9.58E-08		2.12E-07	HP		0
99		rs2057827	16	66,235,992	C	0.165	12M	8	2	6.99E-14	1.17E-08		6.03E-08	HP	3.93E-02	3
100	CYB5B	rs246134	16	69,440,614	T	0.416	12M	5	2	5.22E-10	1.52E-08		2.36E-08	M		0
101	GLP2R-RCVRN*	rs874307	17	9,894,757	G	0.271	12M	6	1	2.65E-09	1.02E-03	5.90E-08	4.13E-05	M	1.25E-03	3
102	MAPT	rs11079727	17	45,899,447	T	0.167	12M	3	2	4.33E-09	5.97E-07		4.97E-05	HP	2.71E-02	3
103		rs7213039	17	51,433,911	T	0.221	12M	4	2	2.90E-08	2.34E-04		8.62E-06	HP	2.43E-02	2
104		rs312750	17	70,347,398	C	0.498	20P	4	2	1.48E-07	3.53E-10		3.93E-06	HB	3.49E-01	1
105	RNF157	rs11539879	17	76,166,467	A	0.043	20P	7	2	1.84E-06	9.30E-09		8.41E-05	HB		0
106	PTPRM	rs1031116	18	7,861,458	T	0.047	12M	3	2	1.98E-08	5.14E-06		1.65E-02	HP	9.86E-02	2
107	MPPE1/GNAL-IMPA2*	rs4797589	18	11,917,411	A	0.45	12M	2	2	6.74E-09	1.04E-06		1.39E-02	HP	7.95E-02	1
108	LDLRAD4	rs7231732	18	13,554,311	C	0.224	20P	7	2	3.62E-14	1.36E-11		3.87E-06	HP/HB	1.17E-01	2
109		rs10048286	18	44,454,280	A	0.192	12M	5	2	1.95E-10	2.24E-09		1.68E-03	HP/HB	2.95E-01	1
110		rs4433895	18	76,663,057	C	0.435	12M	2	2	1.79E-08	1.21E-06		3.05E-05	HP	7.77E-02	1
111	UQCRFS1*	rs10408404	19	29,248,822	A	0.342	12M	3	2	3.05E-08	1.32E-06		2.66E-02	HP	2.70E-01	1
112	ZNF283	rs11673332	19	43,835,290	G	0.12	12M	4	2	5.51E-09	2.12E-05		1.40E-04	HP	2.67E-01	1
113	MICAL3	rs9605473	22	17,980,500	C	0.243	20P	5	2	2.58E-08	1.68E-02		9.76E-03	HP	3.08E-01	1
114		rs8136986	22	47,891,569	A	0.198	12M	4	2	5.31E-10	3.22E-09		7.69E-04	HP/HB		0
115		rs11090806	22	48,002,740	T	0.057	12M	5	1	2.25E-09	1.61E-04	2.14E-07	1.37E-03	HP	3.35E-01	1

^1^ If an index SNP was not within protein coding gene, the closest gene was assigned. Multiple genes were assigned if they were at about the same distance up- and downstream from the index SNP or if the index SNP was within the region of overlapping genes.

^2^ Proxy SNPs with linkage disequilibrium (LD) *r^2^*>70% were excluded. LD with *r^2^*>1% for SNPs within ±1Mb flanking region retained in the table is given in Supplementary Table 2.

^*^ Protein coding genes within ±100 Kb flanking region for the index SNP.

Chr = chromosome; EA = effect allele; EAF = EA frequency.

**Domain** indicates the pleiotropic domain in which minimal GW significant *p*-values were attained in our pleiotropic meta-analysis examining: 12 quantitative markers (12M), 7 diseases and death (8DD), and all 20 phenotypes (20P) domains. *P*-values are given for all pleiotropic meta-tests for a given domain in a given **Pathway** (Fig. 1).

**Group**: the type of an association defined in the “Antagonistic genetic heterogeneity in pleiotropic meta-analysis” section.

***P*_GRASP_**: *p*-values from the pleiotropic meta-analysis of results for the index SNPs reported in GRASP for phenotypes used in 12M or 20P domain reported in column “Domain”. Empty cells: no index SNPs for phenotypes from the corresponding domain (shown in column “Domain”) were reported in GRASP.

**N_P_** and **N_G_** show the number of phenotypes that attained a minimal *p*-value (*p*<0.05) in the meta- or Fisher test in a given phenotypic domain in pathway 1 and Fisher test of GRASP results, respectively.

**P_MOp_**, **P_MOb_**, **P_MFp_**, **P_FcFp_**, **P_OpFc_**, **P_ObFc_**, and **P_FpFc_**: *p*-values for meta-tests (subscripts) defined in Fig. 1.

More details on the associations for these SNPs are given in Supplementary Tables 5 and 6.

**Table 3:** *P*-values from pleiotropic meta-analysis of GRASP results for SNPs within ±1Mb flanking region of the index SNPs not reported in GRASP for 20 selected phenotypes (Table 1).

			**Strongest LD**			**Smallest *p*-value**		
**ID**	**Index SNP**	**Chr**	**Flanking SNP_1_**	**LD**	***P*_GRASP_**	**Flanking SNP_2_**	**LD**	***P*_GRASP_**
3	rs16834550	1	rs2377856	0.557	3.23E-01	rs4949302	0.008	7.03E-06
12	rs1969742	1	rs2777987	1.000	3.27E-01	rs7528684	0.089	3.43E-05
15	rs1934628	1	rs6665563	1.000	4.32E-01	rs6540664	0.000	1.83E-05
27	rs755503	2	rs10186614	0.718	1.81E-01	rs6730157	0.055	1.16E-18
37	rs16829273	3	rs6769314	0.772	1.82E-01	rs9825140	0.000	5.91E-05
40	rs10012631	4	rs1814082	0.266	3.34E-01	rs6831443	0.002	1.04E-03
44	rs1460770	4	rs11098258	0.745	4.14E-01	rs17046113	0.000	5.26E-04
47	rs259130	5	rs259114	0.418	4.01E-01	rs13157900	0.002	1.36E-19
48	rs980831	5	rs17160705	0.196	2.99E-01	rs10037968	0.007	1.21E-04
55	rs6936620	6	rs6457702	0.796	4.09E-01	rs9273363	0.001	1.52E-289
58	rs9482188	6	rs11750990	0.915	2.27E-01	rs1015451	0.420	2.35E-24
60	rs7785072	7	rs12671830	0.322	4.31E-01	rs864745	0.000	7.68E-12
63	rs10953325	7	rs734688	0.480	4.03E-01	rs13245899	0.000	2.74E-22
64	rs7807451	7	rs10224905	0.276	3.78E-01	rs3807989	0.004	5.85E-06
67	rs700278	7	rs696773	0.625	3.69E-01	rs802196	0.108	1.84E-02
71	rs7851478	9	rs16937690	0.745	3.97E-01	rs7022576	0.001	1.23E-05
73	rs2987900	9	rs1536859	0.879	4.34E-01	rs3852402	0.025	2.50E-05
74	rs4149313	9	rs961160	0.005	7.40E-02	rs960644	0.003	8.03E-03
76	rs1476946	9	rs2014343	0.824	3.40E-01	rs7029898	0.000	1.14E-03
81	rs2442100	11	rs2084439	0.762	3.11E-01	rs2517996	0.000	3.82E-03
82	rs11045743	12	rs4149012	0.633	1.45E-01	rs7134375	0.046	9.02E-10
89	rs670676	13	rs682573	0.478	3.86E-01	rs9943924	0.000	9.90E-06
90	rs359412	13	rs359357	0.968	2.54E-01	rs275896	0.000	4.27E-03
91	rs4903274	14	rs11621041	0.807	3.89E-01	rs12433610	0.009	8.04E-04
92	rs4899173	14	rs2127870	0.947	3.73E-01	rs1256507	0.001	4.78E-04
93	rs7151718	14	rs10484104	1.000	4.07E-01	rs1978243	0.001	1.18E-34
96	rs626744	15	rs16962739	0.155	4.09E-01	rs3848128	0.000	1.68E-03
98	rs12444489	16	rs8052106	0.599	2.28E-01	rs3764261	0.000	0.00E+00
100	rs246134	16	rs246141	0.807	4.27E-01	rs9929218	0.000	1.54E-05
105	rs11539879	17	rs1046446	0.369	2.71E-02	rs9892909	0.003	9.29E-06
114	rs8136986	22	rs16997937	0.355	1.59E-01	rs5768709	0.000	1.68E-04

**Chr** = chromosome. **LD**: linkage disequilibrium, *r^2^*.

**Strongest LD**: SNPs with the strongest LD reported in GRASP within ±1Mb flanking region of the index SNPs.

**Smallest *p*-value**: SNPs from the ±1Mb flanking region of the index SNPs attained smallest *p*-values in our pleiotropic meta-analysis of results reported in GRASP.

***P*_GRASP_**: *p*-values from the pleiotropic meta-analysis of the results for flanking SNPs reported in GRASP.

**Figure 2:**
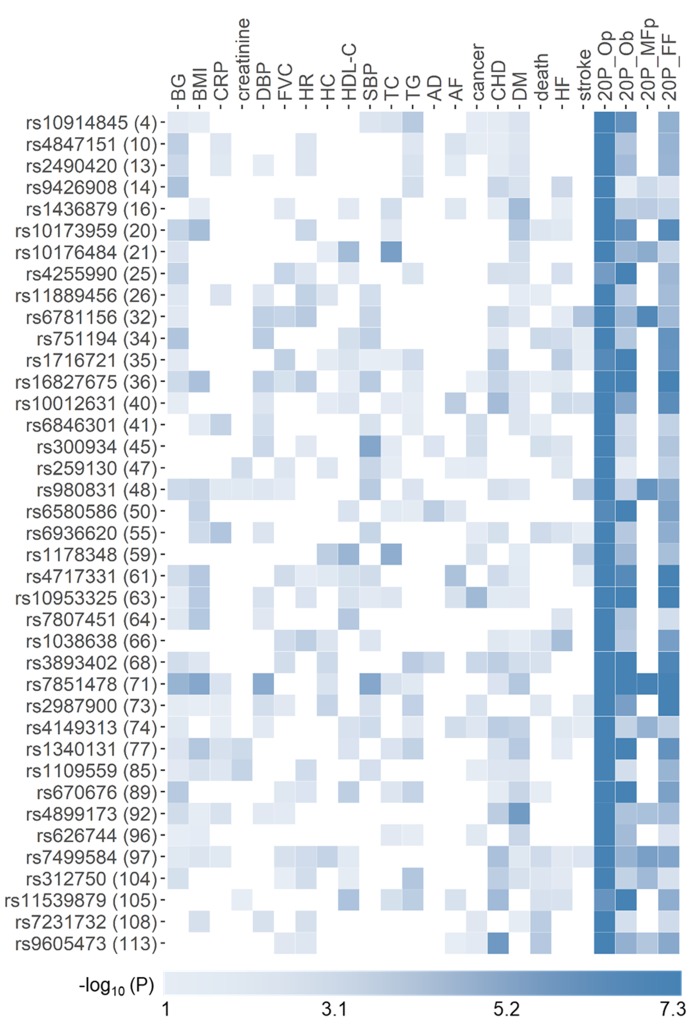
**Heat map of phenotype-specific associations for selected pleiotropic SNPs**. Data are for 39 novel SNPs with pleiotropic associations in the domains of all 20 phenotypes (20P) from Table 2. Numbers in parentheses are SNP IDs in Table 2. Phenotypes are defined in Table 1. FF, Op, and Ob denote pleiotropic meta-tests either from pathway 1 or 2 based on Fisher test, omnibus test with correlation matrix for phenotypes, and omnibus test with correlation matrix for effect statistics (Fig. 1), respectively. MFp denotes pleiotropic meta-tests from pathway 1 (Fig. 1). Colors code -log_10_(*p*-value) trimmed at GW level -log_10_(5×10^-8^)=7.3 for better resolution.

All SNPs attained GW significant associations either in the domain of 12 markers (76 of 115 novel SNPs and seven of nine replicated SNPs) or all 20 phenotypes (39 novel and two replicated SNPs). No associations with *p*<*p_GW_* were identified in the disease/death domain (Supplementary Table 6). The 115 novel and nine replicated SNPs were associated with up to 10 phenotypes at *p*<0.05 (Table 2 and Supplementary Table 4). Figure 2 shows 39 novel pleiotropic SNPs attained GW significance in the domain of 20 phenotypes.

Because *p_pleio_*<*p_GW_*<*p_uni_* for pleiotropic SNPs, this inequality automatically validates pleiotropy for 29 SNPs in pathway 1, as it implies that pleiotropic statistics improved to attain *p_pleio_*<*p_GW_* by pooling contributions from multiple phenotypes with *p_uni_*>*p_GW_* (Supplementary Table 5). Likewise, GW significant pleiotropic associations in meta-analysis in pathway 2 were automatically validated for 37 SNPs with *p_pleio_*>*p_GW_* in each individual cohort (i.e., in pathway 2a) as it implies that pleiotropic statistics improved to attain *p_pleio_*<*p_GW_* in meta-analysis of individual cohorts. For the remaining 49 SNPs in pathway 2, we observed *p_pleio_*<*p_GW_* in at least one cohort. For five of these 49 SNPs, pleiotropic associations did not attain Bonferroni adjusted significance level, *p*<0.05/(N_cohorts_ – 1), in at least one additional cohort, whereas they attained that level for the other 44 SNPs (Supplementary Table 7).

### Antagonistic genetic heterogeneity in pleiotropic meta-analysis

Let us assume that an allele is associated with a higher risk of a phenotype *P_1_* and that *P_1_* is directly correlated with a phenotype *P_2_*, e.g. individuals with larger values of *P_1_* tend to have larger values of *P_2_*. An implicit expectation in medical genetics for partly correlated phenotypes, especially when they are etiologically related, is that the same allele will be also associated with a higher risk of a phenotype *P_2_*. In the framework of the undefined role of evolution in establishing molecular mechanisms of age-related phenotypes (see Introduction), this logic may or may not hold because given allele may not be evolutionary selected in favor or against these phenotypes. A simple approach to examine this logic is to compare the results from different tests used in our pleiotropic meta-analysis. Indeed, the Fisher test in pathways 1b and 2a (Fig. 1) combined *p-*values across phenotypes assuming that they were from independent associations whereas the two omnibus tests adjusted the pleiotropic meta-statistics for correlation among phenotypes ($\Sigma^{P}$-based omnibus test) and the effect statistics ($\Sigma^{B}$-based omnibus test) (see Methods). Accordingly, the differences in *p-*values from the Fisher and omnibus tests reflect the impacts of heterogeneity and/or correlation in genetic associations. Below, we characterize these impacts. We used an *ad-hoc* cut-off for the difference in *p-*values between these tests of ≥2 orders of magnitude to characterize a strong impact.

Given a common assumption that the Fisher pleiotropic meta-test provides inflated *p*-values for correlated phenotypes, correlation-adjusted estimates in the omnibus tests are expected *p_Omnibus_*>*p_Fisher_*. Contrary to this expectation, for most novel SNPs, 93.9% (108 of 115 SNPs) and all nine replicated SNPs, we observe an opposite inequality, i.e., *p_Omnibus_*<*p_Fisher_* by ≥2 orders of magnitude (Table 2 and Supplementary Table 4A). An unconventional set of 108 novel SNPs includes 27 SNPs from HP group in pathway 1, and 54, 5, and 22 SNPs from HP, HB, and HP/HB groups, respectively, in pathway 2 (Table 2). The HP group (27+54=81 SNPs) was characterized by attaining GW significance after the $\Sigma^{P}$-based omnibus tests (i.e., OpFc or MOp, Fig. 1) and substantially larger (≥2 orders of magnitude) *p*-values in the Fisher pleiotropic meta-tests, i.e., *p_MOp_*<*p_MFp_* for pathway 1 and *p_OpFc_*<*p_FpFc_* for pathway 2. The HB group (5 SNPs) resembled the HP group except for having *p_ObFc_*<*p_FpFc_*. The HP/HB group (22 SNPs) was characterized by attaining GW significance after the $\Sigma^{P}$- and $\Sigma^{B}$-based omnibus tests (*p_OpFc,_ p_ObFc_*<*p_FpFc_*) and substantially larger (≥2 orders of magnitude) *p*-values in the Fisher pleiotropic meta-tests (*p_FpFc_*).

**Figure 3:**
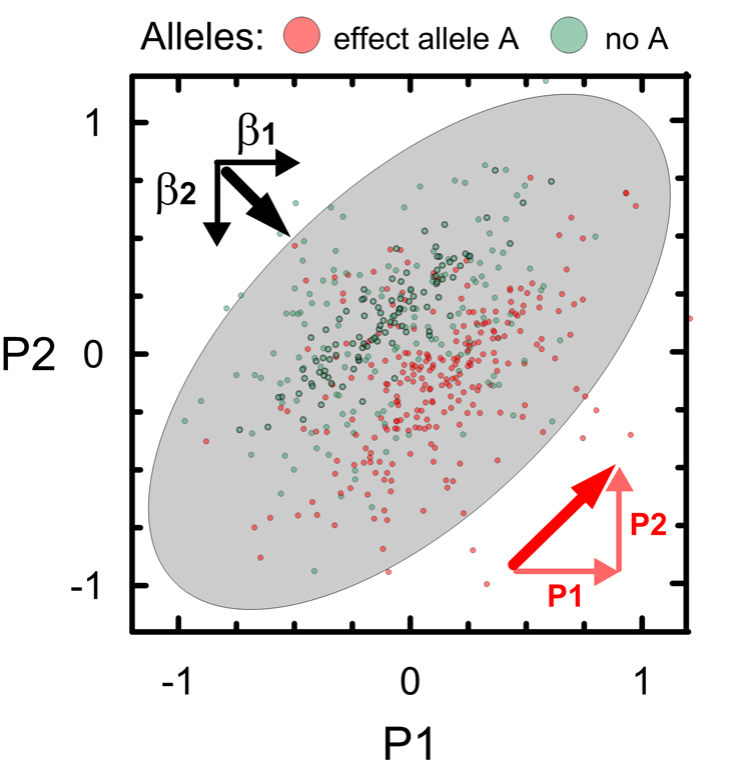
**Schematic illustration of antagonistic genetic heterogeneity in the associations with two partly correlated age-related phenotypes P1 and P2**. Small dots represent a sample of carriers of an effect allele A (red color) and those who do not carry this allele (no A; green color). Ellipse shows correlation of P1 and P2 in this sample (*r*=0.6). Red color denotes vector of correlation of P1 and P2 (thick diagonal vector) and its projections on phenotypes, i.e. **P_1_** (horizontal) and **P_2_** (vertical). Black vectors **β_1_** and **β_2_** denote the effects in the associations of allele A with P1 and P2. Sum of **β_1_** and **β_2_** represents bivariate vector (thick line) of the effects.

These results show that majority of novel SNPs with pleiotropic associations, 93.9%, is characterized by a strong impact of unconventional (natural-selection–free) genetic heterogeneity rather than commonly expected correlation. This form of the natural-selection–free genetic heterogeneity, called antagonistic heterogeneity, is schematically illustrated in Fig. 3 for two partly correlated phenotypes P1 and P2. This heterogeneity results in orthogonal directions of the bivariate vector of genetic effects **β_1_** and **β_2_** for phenotypes P1 and P2 and the vector of the correlation of these phenotypes and, accordingly, in opposite directions of vectors **β_2_** and **P_2_**. As seen from Fig. 3, the antagonistic heterogeneity implies antagonistic directions of genetic effects for directly correlated phenotypes (see Methods).

Pathway 2 provides a natural opportunity to validate the antagonistic heterogeneity in different cohorts. Our analysis shows that this heterogeneity, characterized by *p_OpFc_*, *p_ObFc_*<*p_FpFc_* with a difference of ≥0.2 orders of magnitude for associations with suggestive significance (*p_OpFc_*, *p_ObFc_* <0.1 or *p_FpFc_*<0.1), was replicated for 77 of 81 novel pleiotropic SNPs in two or more cohorts (Supplementary Table 8).

Lastly, the M group (7 SNPs) included 2 SNPs from pathway 1 and 5 SNPs from pathway 2. It was characterized by attaining GW significance in several tests and/or minor (<2 orders of magnitude) differences between *p*-values in OpFc and FpFc tests (pathway 2) and MOp and MFp (pathway 1).

### Bioinformatics analysis

**Figure 4:**
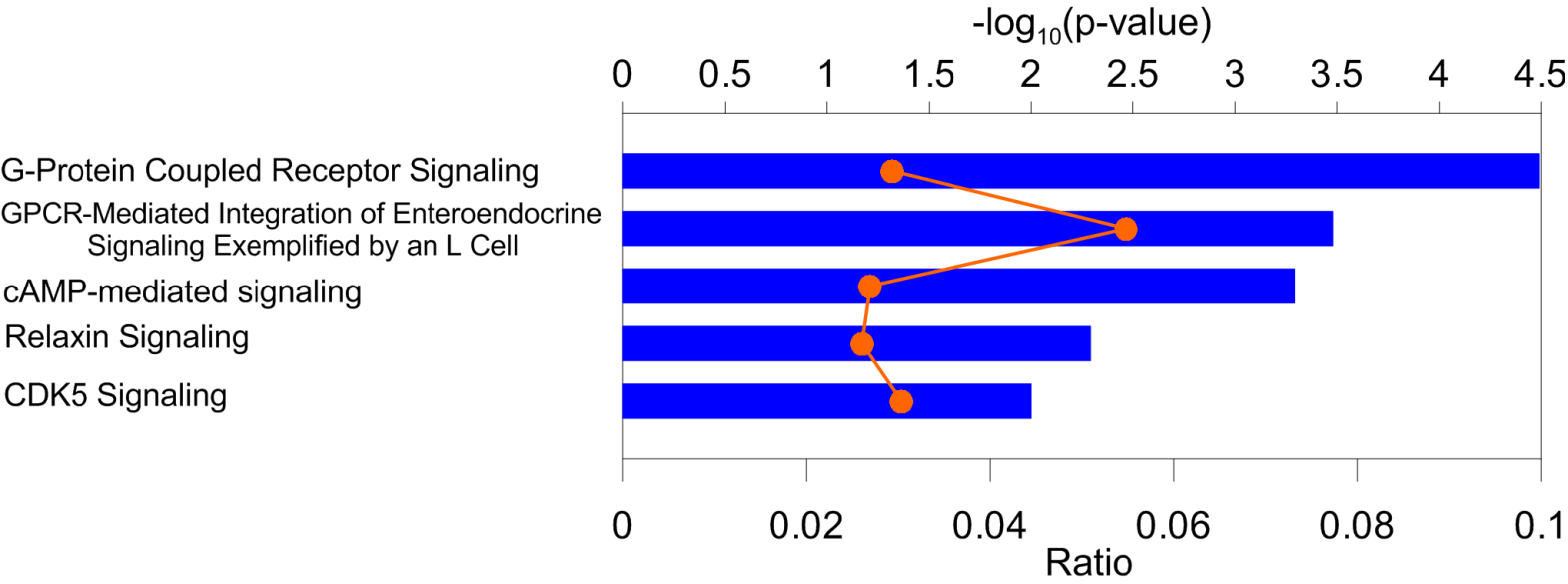
**Enrichment of pathways in the Ingenuity Pathway Analysis (IPA) bioinformatics tool**. Blue bars (upper x-axis) show –log_10_(*p*-value) for the top five IPA pathways. Proportion of genes from the identified sets to those in the IPA pathways (orange symbols and line) is shown on the lower *x*-axis. Numerical estimates are given Supplementary Table 10.

We performed biological pathway (IPA, www.qiagenbioinformatics.com) and gene ontology (GO) biological processes (BPs) (DAVID [24]) enrichment analysis for 96 protein coding genes mapped from 108 pleiotropic SNPs (Table 2). We excluded genes for five non-validated SNPs (Table 2, ID 1, 24, 25, 49, and 95) and for two SNPs (Table 2, ID 53, 55) from the Major Histocompatibility Complex (MHC). The GO analysis (by DAVID 6.8 GO category “GO_direct”) identified 10 BPs with enrichment for genes at *p*<0.05 (Fisher’s exact test) (Supplementary Table 9). Two specific terms, *adenylate cyclase-activating G-protein coupled receptor signaling pathway* and *activation of adenylate cyclase activity*, were enriched at *p*<10^-4^ and *p*<10^-3^, respectively. The IPA analysis identified 18 pathways (Supplementary Table 10) enriched for genes at *p*<0.05 (Fisher’s exact test). The strongest enrichment was observed for *G-protein coupled receptor signaling* (*p*<10^-4^), *GPCR-mediated integration of enteroendocrine signaling exemplified by an L cell* and *cAMP-mediated signaling* (*p*<10^-3^) (Figure 4) that is consistent with enrichment of GO BPs.

## DISCUSSION

**Figure 5:**
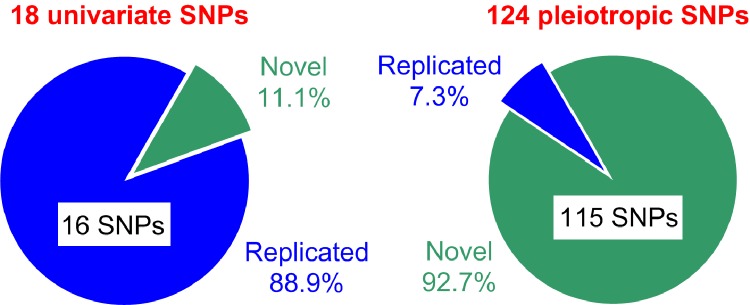
**Summary of SNPs identified in the univariate and pleiotropic meta-analyses**. Green and blue colors denote novel and replicated SNPs, respectively.

Our univariate and pleiotropic meta-analyses of 20 age-related phenotypes dealing with the natural-selection–free genetic heterogeneity identified large number of non-proxy SNPs, 142 SNPs, with GW significance in a relatively modest sample of 33,431 individuals of Caucasian ancestry from five longitudinal studies (Figure 5). Only 18 SNPs (12.7%) were identified in the univariate meta-analysis with most SNPs, 88.9% (16 of 18 SNPs), replicating previously reported associations, primarily with lipids. Two novel SNPs were associated with HC (rs6745983 in *TMEM163* gene) and BG (rs10885409 in *TCF7L2* gene). Two of 16 replicated SNPs, rs780094 (*GCKR* gene) and rs261332 (*LIPC/LIPC-AS1* genes) associated with BG and TC, respectively, showed evidences for inter-cohort heterogeneity in the effect sizes. Accordingly, smaller *p*-values were attained by addressing this heterogeneity. The association of rs2155216 (*BUD13-ZPR1/APOA5* gene locus) with TG was replicated with substantially higher (4-fold) efficiency in our analysis than in previous study [22] that was achieved, in part, by leveraging information on repeated measurements from longitudinal follow up.

Most SNPs, 87.3% (124 of 142 SNPs), were identified in our pleiotropic meta-analyses and most of them, 92.7% (115 of 124 SNPs), were novel (Figure 5). Novel pleiotropic SNPs attained GW significance by combining associations with multiple phenotypes, which individually did not attain GW significance in univariate meta-analysis (Figure 2). Therefore, pleiotropic meta-analysis has power to identify SNPs leveraging signals, which are often considered as noise in univariate GWAS. Most importantly, our analysis showed that the associations for most novel pleiotropic SNPs, 93.9% or 108 of 115 SNPs (and all 9 replicated SNPs) were strongly affected by the natural-selection–free genetic heterogeneity in a rarely recognized form of antagonistic heterogeneity, implying antagonistic directions of genetic effects for directly correlated phenotypes. This heterogeneity has not been addressed in the currently prevailing GWAS.

Thus, our analysis provides compelling evidences that the traditional sample-size-centered GWAS approach is well adapted to handle homogeneous univariate associations (one SNP – one phenotype) within Mendelian framework whereas most associations with age-related phenotypes are more complex and well beyond that framework. This complexity substantially decreases the efficiency of the analysis of such phenotypes within the current GWAS framework. This conclusion leads to three major implications.

First, discovery of a large number of common genetic variants associated with different phenotypes in GWAS [25], raised concern known as a “missing heritability problem” [26]. The problem is that the estimated variance attributed to GWAS-identified common SNPs is only a fraction of the expected genetic variance from the estimates of heritability of a phenotype. This problem even leads to questioning whether genetics could be helpful for improving health care [27]. Our results show that in large part missing heritability problem for age-related phenotypes can be due to missing associations with complex genetic predisposition to these phenotypes.

Second, the traditional GWAS approach is built on the concept of a “true” or causal genetic effect (see the Introduction) whereas biologically plausible concept for age-related phenotypes is that based on the undefined role of evolution in establishing their molecular mechanisms. The letter implies that the same genetic variant can predispose differently to the same phenotype in different population groups, even of the same ancestry, e.g., throughout the life course and/or across generations. This concept particularly implies that “small” genetic effects (often expected in GWAS of age-related phenotypes) can be due to a complex superposition of large effects rather than small penetrance. Then, just relaying on large samples in GWAS of age-related phenotypes without dissecting the natural-selection–free genetic heterogeneity is inefficient because increasing the sample size will also increase heterogeneity [28]. The current study supports this concept by highlighting strong impact of antagonistic heterogeneity, which is counter-intuitive in medical genetics but natural within the evolutionary framework. The antagonistic heterogeneity is also supported by the analyses of alleles from well-known apolipoprotein B and E genes [16,17].

Third, all pleiotropic SNPs attained GW significance in the two largest domains of 12 quantitative markers and all 20 phenotypes. These findings support the hypothesis that pleiotropic predisposition to age-related phenotypes can be driven by fundamental mechanisms associated with aging [8,18,19,29]. This attractive hypothesis in gerontology conceptualized as geroscience [28,30] is based on observations that aging-related processes [31,32] are among the most important risk factors for geriatric diseases of distinct etiologies. The dominant role of antagonistic heterogeneity in pleiotropic associations cautions against simplistic approaches in studies of pleiotropic effects on age-related phenotypes and emphasizes the importance of personalized medicine which can potentially handle complexity of risk profiles on an individual basis [15,33,34]. Dissecting the role of antagonistic heterogeneity is particularly important as this provides a genetic basis for strategies focusing on anti-side-effect treatments in medical care.

Finally, our bioinformatics analysis of genes for pleiotropic SNPs identified three interlinked pathways, G*-protein coupled receptor signaling, GPCR-mediated integration of enteroendocrine signaling exemplified by an L cell*, and *cAMP-mediated signaling*. G-protein coupled receptors (GPCRs), members of the largest family of membrane proteins, are activated by a wide variety of external signals (e.g., light, odorants, hormones, neurotransmitters, pharmacologic agents, etc.) and modulate the activity of signaling pathways involved in most physiological processes. In particular, GPCRs partly mediate secretion of enteroendocrine peptide hormones glucagon-like peptide 1 (GLP-1) and peptide YY (PYY) by L-cells, relevant to food intake and blood glucose homeostasis [35,36]. GPCRs play a causative role in many human diseases and are the important drug targets [37,38]. Adenylyl cyclase, a membrane-associated enzyme, when activated by G proteins, catalyzes synthesis of important second messenger cyclic adenosine monophosphate (cAMP). cAMP is involved in diverse biological processes (e.g., glycogen metabolism, hormone regulation, gene expression, sensory signal transduction). The cAMP levels also partly mediate secretion of enteroendocrine peptide hormones linked to feeding/metabolic control [35] and are associated with immune function [39]. Top GO BPs (*adenylate cyclase-activating G-protein coupled receptor signaling pathway* and *activation of adenylate cyclase activity*) support an enrichment of BPs related to GPCRs function and cAMP synthesis. Thus, these results suggest an important role of G protein-dependent signaling and adenylyl cyclase activity necessary for the proper biological response of cells to hormones and other extracellular signals in pleiotropic effect on age-related phenotypes.

## METHODS

### Study cohorts

Data were drawn from the Atherosclerosis Risk in Communities (ARIC) study [40,41], the Cardiovascular Health Study (CHS) [42], the Multi-Ethnic Study of Atherosclerosis (MESA) [43], the Framingham Heart Study (FHS) [44-46], and the Health and Retirement Study (HRS) [47] for individuals who identified themselves as of Caucasian ancestry.

### Phenotypes

The analyses focused on 20 phenotypes (12 quantitative markers, 7 diseases, and death) listed in Table 1. Given longitudinal design of the studied cohorts, the analyses leveraged repeated measurements of quantitative markers during follow up and timing information on onsets of diseases or death (Supplementary Table 1). These not strongly correlated phenotypes (Supplementary Figure 1) were available in majority of the selected cohorts. All studies except HRS collected information on diseases and death in population samples during follow-up. The HRS assessed information on date of death from the National Death Index Cause of Death file. Age at onset of diseases in HRS was assessed from the linked Medicare service use files, which include enrollment information and the diagnoses made (International Classification of Disease-revision 9, Clinical Modification) during episodes of care paid for by the Medicare system.

### Genotyping

SNPs were available from Affymetrix 1M chip in ARIC and MESA, Illumina CVDSNP55v1_A chip (~50K SNPs) in FHS and CHS cohorts, Illumina HumanCNV370v1 chip (370K SNPs) in CHS, Affymetrix 500K in FHS, and Illumina HumanOmni 2.5 Quad chip (~2.5M SNPs) in HRS. SNPs were included in the analyses after quality control in each study (call rate>95%, Hardy-Weinberg disequilibrium *p*<10^-6^, minor allele frequency [MAF] >2%). In case of marginally smaller MAF for prioritized SNPs in a specific cohort compared to the MAF cut off, these SNPs were used regardless of the MAF cut off. To facilitate cross-platform comparisons, we selected directly genotyped (index) SNPs using all available arrays for each study. Non-genotyped SNPs were imputed (IMPUTE2 [48],) according to the 1000 Genomes Project Phase I integrated variant set release (SHAPEIT2) in the NCBI build 37 (hg19) coordinate. Only SNPs with high imputation quality (info>0.8) were retained for the analyses.

### Mapping to genes

SNPs were mapped to genes using variant effect predictor from Ensembl and NCBI SNP database (assembly GRCh38.p7). If an index SNP was not within protein coding gene, the closest gene(s) within ±100 Kb flanking region was (were) assigned. Multiple genes were selected if they were at about the same distance up- and downstream from the index SNP or the index SNP was within the region of overlapping genes.

### Selecting SNPs in univariate GWAS in stage 1

GWAS was performed for each phenotype in each cohort separately using *plink* software [21]. In this analysis, we used quantitative markers measured at baseline, and diseases and death as binary outcomes. An additive genetic model with minor allele as an effect allele was adopted in all analyses throughout this article. We computed pleiotropic *p*-value by combining *p*-values for individual markers and binary outcomes for SNPs attained nominal significance only (*p*<0.05) using Fisher’s test [49]. This computationally efficient but overly-simplified approach was used for prioritizing of promising SNPs. Because this procedure provides inflated *p*-values, we selected top 1,000 SNPs (with smallest *p*-values) for all downstream analyses, rather than selecting SNPs based on specific cut off for pleiotropic *p*-values.

### Leveraging longitudinal information in stage 1 analysis

The analysis of the selected 1,000 SNPs were enhanced by leveraging longitudinal information. For quantitative markers, we used all measurements available during follow up of the same individuals. Information on longitudinal measurements has multiple advantages including potential gain in statistical power in the analyses [50]. To correct for repeated-measurements (all cohorts) and familial (FHS) correlations in the analyses of quantitative markers, we mostly used the linear mixed effects model (*lme4* package in R [51]). Measurements of BG, BMI, HDL-C, HR, TC, and TG were natural-log-transformed to offset potential bias due to skewness of their frequency distributions. They were multiplied by 100 for better resolution. Measurements of creatinine, DBP, FVC, HC and SBP were used in their natural scale as no significant skewness was observed. Given gamma-like frequency distributions of CRP, a generalized linear mixed model (glmmPQL package in R) with a gamma function and log-link was used. We evaluated the associations for SNPs given the measurements of quantitative markers for individuals of a given age at each examination with available measurements.

Longitudinal information on time to events was implemented in the Cox proportional hazards mixed effects model (*coxme* package in R). This model addressed familial relatedness. In the FHS, we used both prospective and retrospective onsets. The use of retrospective onsets in a failure-type model is justified by Prentice, Breslow [52]. These analyses provide estimates of the effects in a given population. Time variable in these analyses was the age at onset of an event or at right censoring.

The models were adjusted for: (all studies) age and sex; (ARIC, CHS, and MESA) field center; (HRS) HRS cohorts, and (FHS) whether the DNA samples had been subject to whole-genome amplification [53]. No adjustment for principal components was performed as argued in Ref [54].

### Meta-analyses in stage 2

After stage 1, for each SNP we have a table with the association statistics for up to 20 phenotypes in 5 cohorts. These statistics were combined along two possible pathways: (i) first across studies and then across phenotypes and (ii) first across phenotypes and then across studies. To deal with the natural-selection–free heterogeneity in genetic predisposition to age-related phenotypes (see below), we used four tests in pathway 1 and three tests in pathway 2 (Fig. 1).

In pathway 1, univariate (phenotype-specific) meta-analysis combining the stage 1 statistics for the selected SNPs across cohorts (pathway 1a) was performed using the Fisher test and the traditional GWAS fixed-effects meta-test. Pleiotropic meta-analysis (pathway 1b) was performed by combining the univariate meta-statistics for the same SNPs across phenotypes. In pathway 1b, we used the Fisher test and two omnibus tests. The latter were designed to address correlations between the effect statistics and phenotypes. In pathway 2, we performed first pleiotropic meta-analysis by combining the stage 1 univariate statistics across phenotypes in each cohort separately (pathway 2a) using the Fisher test and the two omnibus tests as in pathway 1b. Then, we combined these pleiotropic meta-statistics across cohorts using the Fisher test (pathway 2b) (details on all tests are below).

### Fixed-effects meta-test

We adopted a conventional GWAS meta-test using a fixed effects model with inverse-variance weighting (METAL software [55]). This test combines the estimated effect sizes across cohorts for each phenotype. Combining effect sizes is feasible because each phenotype was harmonized to be on the same scale and unit across cohorts. This meta-test could account for the directions of effects in different cohorts, and is more powerful than those tests that combines *p*-values or Z-scores [56]. In pathway 1a, the weighted average of the effect sizes was calculated as $\Sigma_{j}({\hat{w}}_{j}{\hat{\beta }}_{j})/\Sigma_{j}({\hat{w}}_{j})$ with variance 1/$\Sigma_{j}({\hat{w}}_{j})$, where ${\hat{w}}_{j}$ is the inverse variance of effect size ${\hat{\beta }}_{j}\;$in the cohort $j\in \left(\overset{-}{1,5}\right)$ for a given phenotype and SNP. Wald test was then used to obtain *p*-value.

### Fisher’s test

The Fisher method [49] combines *p-*values assuming that they are from independent tests. In pathway 1a, this test combines *p*-values across cohorts. It has power to reject the “null” hypothesis of no pooled effect regardless of the effect sizes and directions in the cohort-specific estimates. Accordingly, the Fisher test can indicate heterogeneity in genetic associations by providing smaller *p-*value than that from the fixed-effects meta-test. In pathways 1b and 2a, the Fisher test combines *p*-values across phenotypes. This test is often used for pleiotropic meta-analysis of modestly correlated phenotypes [57]. Because the Fisher method combines *p*-values from multiple tests, it addresses the problem of multiple testing by increasing the number of degrees of freedom.

### Omnibus tests

The statistics from tests of the same SNPs with different phenotypes may or may not be independent. It is then argued that tests penalizing for correlation of such statistics should be used to deflate the Fisher test estimates. A commonly adopted test in this case is an omnibus test [58-60].

For a certain SNP we have an estimated effect size ${\hat{\beta }}_{ij}$ and its standard error ${\hat{\sigma }}_{ij}$ for the phenotype $i\in \left({\overset{-}{1,K}}_{j}\right)$ in the cohort $j\in \left(\overset{-}{1,5}\right)$, where $K_{j}$ is the number of phenotypes in study j. The omnibus test statistic is constructed as z^'jΣj-1z^j where ${\hat{z}}_{j}={\hat{\beta }}_{j}/{\hat{\sigma }}_{j}$ is a *z*-score vector of associations of SNPs with phenotypes and $\Sigma_{j}$ is the correlation matrix of the *z*-scores to be estimated [58,59]. Accordingly, this test takes into account the correlation of the effect statistics for different phenotypes. Under the null hypothesis ($\beta_{j}=0$), the test statistic follows a chi-squared distribution with $K_{j}$ degrees of freedom

z^'jΣj-1z^j~χKj2 ,

from which we obtained a combined *p-*value $p_{j}$ in the study j. Accordingly, this statistic addresses the problem of multiple testing by increasing the number of degrees of freedom.

Correlation matrices Σ were estimated based on the effect statistics of simulated SNPs in association models with phenotypes (denoted $\Sigma \equiv \Sigma^{B}$). We randomly permuted the dosage data for alleles of a given SNP for 250 times, used each permuted SNP in association models with each of *K* phenotypes, and estimated $\Sigma^{B}$ based on the 250 effect sizes of permuted SNPs with *K* phenotypes. Likewise, Σ can be also constructed by evaluating the correlations between phenotypes [61] (denoted $\Sigma \equiv \Sigma^{P}$**)**. The correlation matrices, $\Sigma^{B}$ and $\Sigma^{P}$**,** were constructed for pathways 1 and 2 (Fig. 1) separately. Cohort-specific matrices $\Sigma_{j}^{B}$ for pathway 2 were constructed by evaluating correlations of the effect statistics in each cohort. For pathway 1, matrix $\Sigma_{m}^{B}$ was constructed by evaluating correlations of the effect statistics from a fixed effect meta-test of all cohorts for each permutation. The phenotype-based cohort-specific matrices for pathway 2, $\Sigma_{j}^{P}$, were evaluated using phenotype measurements in each cohort separately and matrix for pathway 1, $\Sigma_{m}^{P}$, was evaluated using phenotype measurements in the combined data from all cohorts. All correlation matrices were constructed using averaged values for quantitative traits measured longitudinally at different visits.

### Correlation and genetic heterogeneity

Omnibus tests are commonly used to penalize correlation between the effect statistics or phenotypes in pleiotropic meta-analysis. This can be explicitly illustrated by the test statistics z^'jΣj-1z^j in two-dimensional case,

z^'jΣj-1z^j=z^12Σ22-z^1z^2Σ21+z^22Σ11-z^1z^2Σ12/det(Σ) . (1)

Either the $\Sigma^{B}$ or $\Sigma^{P}$-based omnibus test (see above) penalize pleiotropic meta-statistics, if ${{\hat{z}}_{1}{\hat{z}}_{2}\Sigma }_{21},{{\hat{z}}_{1}{\hat{z}}_{2}\Sigma }_{12}>0$.

The undefined role of evolution in establishing molecular mechanisms of age-related phenotypes is the source of genetic heterogeneity, which is driven by the lack, rather than the presence, of the evolutionary pressure in favor or against these phenotypes. The natural-selection–free genetic heterogeneity suggests that mechanisms driving associations of genes with age-related phenotypes and correlation between these phenotypes are, generally, of different origins. In case of antagonistic genetic heterogeneity (see “Antagonistic genetic heterogeneity in pleiotropic meta-analysis” section), omnibus test provides smaller *p-*values compared to the Fisher test as can be seen from (1) because ${{\hat{z}}_{1}{\hat{z}}_{2}\Sigma }_{21},{{\hat{z}}_{1}{\hat{z}}_{2}\Sigma }_{12}<0$ in this case.

### Significance and novelty

The fixed-effect, Fisher, and two omnibus meta-tests used in our analyses have power to identify associations, adjusted and unadjusted for correlation and heterogeneity in genetic predisposition to age-related phenotypes (see above). Fisher and two omnibus tests used in pleiotropic meta-analysis address the problem of multiple testing by increasing the number of degrees of freedom. Accordingly, GW level (*p*=5×10^-8^) attained in either of these tests was used as a cut off for the significance for a given SNP. The difference between *p*-values from these tests for a given SNP was used to characterize the impact of correlation and heterogeneity on the association.

SNPs were considered as novel if they attained GW significance in: (i) our univariate meta-analysis but were not reported in GRASP catalog [62] at *p*≤5×10^-8^ or (ii) our pleiotropic meta-analysis but not in our pleiotropic analysis of the results collected in GRASP. For univariate meta-analysis, we used evidences for the same (index) SNP in our study and GRASP. For pleiotropic meta-analysis, we selected associations from GRASP for the index SNPs with 20 phenotypes used in our analysis (Table 1). If an index SNP was not available in GRASP for the selected phenotypes, we selected SNPs within ±1Mb flanking region. Then we performed pleiotropic meta-analysis by applying the Fisher method to these GRASP results to present evidence for pleiotropy in prior studies. We used the Fisher statistics *p_grasp_* because the effect sizes were not reported in GRASP. We used a flat *p*-value, *p*=0.4, to penalize the Fisher statistics for phenotypes with *p-*values not reported in GRASP. For flanking SNPs, we reported associations for SNPs having: (i) the strongest LD with the index SNP and (ii) the smallest *p_grasp_* for SNPs within ±1Mb flanking region.

### Heterogeneity coefficient

We used METAL software [55] to evaluate the heterogeneity coefficient I^2^. The I^2^ can be interpreted as the percentage of the total variability in a set of effect sizes due to between-sample variability.

## Supplementary Material

Supplementary File

Supplementary Table 3

Supplementary Table 4

Supplementary Table 5

Supplementary Table 6

Supplementary Table 7

Supplementary Table 8

Supplementary Table 9

Supplementary Table 10

## References

[1] Button KS, Ioannidis JP, Mokrysz C, Nosek BA, Flint J, Robinson ES, Munafò MR. Power failure: why small sample size undermines the reliability of neuroscience. Nat Rev Neurosci. 2013; 14:365–76. 10.1038/nrn3475 23571845

[2] Yang J, Zeng J, Goddard ME, Wray NR, Visscher PM. Concepts, estimation and interpretation of SNP-based heritability. Nat Genet. 2017; 49:1304–10. 10.1038/ng.3941 28854176

[3] Hamosh A, Scott AF, Amberger JS, Bocchini CA, McKusick VA. Online Mendelian Inheritance in Man (OMIM), a knowledgebase of human genes and genetic disorders. Nucleic Acids Res. 2005; 33:D514–17. 10.1093/nar/gki033 15608251 PMC539987

[4] Plomin R, Haworth CM, Davis OS. Common disorders are quantitative traits. Nat Rev Genet. 2009; 10:872–78. 10.1038/nrg2670 19859063

[5] Lewontin RC. Annotation: the analysis of variance and the analysis of causes. Am J Hum Genet. 1974; 26:400–11. 4827368 PMC1762622

[6] Rose SP. Commentary: heritability estimates--long past their sell-by date. Int J Epidemiol. 2006; 35:525–27. 10.1093/ije/dyl064 16645027

[7] Nesse RM, Ganten D, Gregory TR, Omenn GS. Evolutionary molecular medicine. J Mol Med (Berl). 2012; 90:509–22. 10.1007/s00109-012-0889-9 22544168 PMC4416654

[8] Kirkwood TB, Cordell HJ, Finch CE. Speed-bumps ahead for the genetics of later-life diseases. Trends Genet. 2011; 27:387–88. 10.1016/j.tig.2011.07.001 21824675

[9] Oeppen J, Vaupel JW. Demography. Broken limits to life expectancy. Science. 2002; 296:1029–31. 10.1126/science.1069675 12004104

[10] Corella D, Ordovás JM. Aging and cardiovascular diseases: the role of gene-diet interactions. Ageing Res Rev. 2014; 18:53–73. 10.1016/j.arr.2014.08.002 25159268

[11] Kulminski AM. Unraveling genetic origin of aging-related traits: evolving concepts. Rejuvenation Res. 2013; 16:304–12. 10.1089/rej.2013.1441 23768105 PMC3746287

[12] Vijg J, Suh Y. Genetics of longevity and aging. Annu Rev Med. 2005; 56:193–212. 10.1146/annurev.med.56.082103.104617 15660509

[13] Crespi B, Stead P, Elliot M. Evolution in health and medicine Sackler colloquium: comparative genomics of autism and schizophrenia. Proc Natl Acad Sci USA. 2010 (Suppl 1); 107:1736–41. 10.1073/pnas.0906080106 19955444 PMC2868282

[14] Day-Williams AG, Zeggini E. The effect of next-generation sequencing technology on complex trait research. Eur J Clin Invest. 2011; 41:561–67. 10.1111/j.1365-2362.2010.02437.x 21155765 PMC3107422

[15] Ukraintseva S, Yashin A, Arbeev K, Kulminski A, Akushevich I, Wu D, Joshi G, Land KC, Stallard E. Puzzling role of genetic risk factors in human longevity: “risk alleles” as pro-longevity variants. Biogerontology. 2016; 17:109–27. 10.1007/s10522-015-9600-1 26306600 PMC4724477

[16] Kulminski AM, Kernogitski Y, Culminskaya I, Loika Y, Arbeev KG, Bagley O, Duan M, Arbeeva L, Ukraintseva SV, Wu D, Stallard E, Yashin AI. Uncoupling associations of risk alleles with endophenotypes and phenotypes: insights from the ApoB locus and heart-related traits. Aging Cell. 2017; 16:61–72. 10.1111/acel.12526 27683205 PMC5242299

[17] Kulminski AM, Raghavachari N, Arbeev KG, Culminskaya I, Arbeeva L, Wu D, Ukraintseva SV, Christensen K, Yashin AI. Protective role of the apolipoprotein E2 allele in age-related disease traits and survival: evidence from the Long Life Family Study. Biogerontology. 2016; 17:893–905. 10.1007/s10522-016-9659-3 27447179 PMC5065761

[18] Goh KI, Cusick ME, Valle D, Childs B, Vidal M, Barabási AL. The human disease network. Proc Natl Acad Sci USA. 2007; 104:8685–90. 10.1073/pnas.0701361104 17502601 PMC1885563

[19] Kulminski AM, He L, Culminskaya I, Loika Y, Kernogitski Y, Arbeev KG, Loiko E, Arbeeva L, Bagley O, Duan M, Yashkin A, Fang F, Kovtun M, et al Pleiotropic Associations of Allelic Variants in a 2q22 Region with Risks of Major Human Diseases and Mortality. PLoS Genet. 2016; 12:e1006314. 10.1371/journal.pgen.1006314 27832070 PMC5104356

[20] Barabási AL, Gulbahce N, Loscalzo J. Network medicine: a network-based approach to human disease. Nat Rev Genet. 2011; 12:56–68. 10.1038/nrg2918 21164525 PMC3140052

[21] Purcell S, Neale B, Todd-Brown K, Thomas L, Ferreira MA, Bender D, Maller J, Sklar P, de Bakker PI, Daly MJ, Sham PC. PLINK: a tool set for whole-genome association and population-based linkage analyses. Am J Hum Genet. 2007; 81:559–75. 10.1086/519795 17701901 PMC1950838

[22] Teslovich TM, Musunuru K, Smith AV, Edmondson AC, Stylianou IM, Koseki M, Pirruccello JP, Ripatti S, Chasman DI, Willer CJ, Johansen CT, Fouchier SW, Isaacs A, et al Biological, clinical and population relevance of 95 loci for blood lipids. Nature. 2010; 466:707–13. 10.1038/nature09270 20686565 PMC3039276

[23] Kulminski AM, Loika Y, Culminskaya I, Arbeev KG, Ukraintseva SV, Stallard E, Yashin AI. Explicating heterogeneity of complex traits has strong potential for improving GWAS efficiency. Sci Rep. 2016; 6:35390. 10.1038/srep35390 27739495 PMC5064392

[24] Huang W, Sherman BT, Lempicki RA. Systematic and integrative analysis of large gene lists using DAVID bioinformatics resources. Nat Protoc. 2009; 4:44–57. 10.1038/nprot.2008.211 19131956

[25] Welter D, MacArthur J, Morales J, Burdett T, Hall P, Junkins H, Klemm A, Flicek P, Manolio T, Hindorff L, Parkinson H. The NHGRI GWAS Catalog, a curated resource of SNP-trait associations. Nucleic Acids Res. 2014; 42:D1001–06. 10.1093/nar/gkt1229 24316577 PMC3965119

[26] Maher B. Personal genomes: the case of the missing heritability. Nature. 2008; 456:18–21. 10.1038/456018a 18987709

[27] Goldstein DB. Common genetic variation and human traits. N Engl J Med. 2009; 360:1696–98. 10.1056/NEJMp0806284 19369660

[28] Franceschi C, Garagnani P. Suggestions from Geroscience for the Genetics of Age-Related Diseases. PLoS Genet. 2016; 12:e1006399. 10.1371/journal.pgen.1006399 27832069 PMC5104376

[29] Franco OH, Karnik K, Osborne G, Ordovas JM, Catt M, van der Ouderaa F. Changing course in ageing research: the healthy ageing phenotype. Maturitas. 2009; 63:13–19. 10.1016/j.maturitas.2009.02.006 19282116

[30] Kaeberlein M, Rabinovitch PS, Martin GM. Healthy aging: the ultimate preventative medicine. Science. 2015; 350:1191–93. 10.1126/science.aad3267 26785476 PMC4793924

[31] Guarente L, Franklin H. Franklin H. Epstein Lecture: Sirtuins, aging, and medicine. N Engl J Med. 2011; 364:2235–44. 10.1056/NEJMra1100831 21651395

[32] Zhavoronkov A, Moskalev A. Editorial: Should We Treat Aging as a Disease? Academic, Pharmaceutical, Healthcare Policy, and Pension Fund Perspectives. Front Genet. 2016; 7:17. 10.3389/fgene.2016.00017 26909101 PMC4754422

[33] Schork NJ. Personalized medicine: time for one-person trials. Nature. 2015; 520:609–11. 10.1038/520609a 25925459

[34] Jazwinski SM, Kim S, Dai J, Li L, Bi X, Jiang JC, Arnold J, Batzer MA, Walker JA, Welsh DA, Lefante CM, Volaufova J, Myers L, et al and Georgia Centenarian Study and the Louisiana Healthy Aging Study. HRAS1 and LASS1 with APOE are associated with human longevity and healthy aging. Aging Cell. 2010; 9:698–708. 10.1111/j.1474-9726.2010.00600.x 20569235 PMC2941558

[35] Spreckley E, Murphy KG. The L-Cell in Nutritional Sensing and the Regulation of Appetite. Front Nutr. 2015; 2:23. 10.3389/fnut.2015.00023 26258126 PMC4507148

[36] Reimann F, Tolhurst G, Gribble FM. G-protein-coupled receptors in intestinal chemosensation. Cell Metab. 2012; 15:421–31. 10.1016/j.cmet.2011.12.019 22482725

[37] Zalewska M, Siara M, Sajewicz W. G protein-coupled receptors: abnormalities in signal transmission, disease states and pharmacotherapy. Acta Pol Pharm. 2014; 71:229–43. 25272642

[38] Schöneberg T, Schulz A, Biebermann H, Hermsdorf T, Römpler H, Sangkuhl K. Mutant G-protein-coupled receptors as a cause of human diseases. Pharmacol Ther. 2004; 104:173–206. 10.1016/j.pharmthera.2004.08.008 15556674

[39] Serezani CH, Ballinger MN, Aronoff DM, Peters-Golden M. Cyclic AMP: master regulator of innate immune cell function. Am J Respir Cell Mol Biol. 2008; 39:127–32. 10.1165/rcmb.2008-0091TR 18323530 PMC2720142

[40] Sharrett AR. The Atherosclerosis Risk in Communities (ARIC) Study. Introduction and objectives of the hemostasis component. Ann Epidemiol. 1992; 2:467–69. 10.1016/1047-2797(92)90096-9 1342297

[41] Investigators TA. The Atherosclerosis Risk in Communities (ARIC) Study: design and objectives. The ARIC investigators. Am J Epidemiol. 1989; 129:687–702. 10.1093/oxfordjournals.aje.a115184 2646917

[42] Fried LP, Borhani NO, Enright P, Furberg CD, Gardin JM, Kronmal RA, Kuller LH, Manolio TA, Mittelmark MB, Newman A, O’Leary DH, Psaty B, Rautaharju P, et al The Cardiovascular Health Study: design and rationale. Ann Epidemiol. 1991; 1:263–76. 10.1016/1047-2797(91)90005-W 1669507

[43] Bild DE, Bluemke DA, Burke GL, Detrano R, Diez Roux AV, Folsom AR, Greenland P, Jacob DR Jr, Kronmal R, Liu K, Nelson JC, O’Leary D, Saad MF, et al Multi-Ethnic Study of Atherosclerosis: objectives and design. Am J Epidemiol. 2002; 156:871–81. 10.1093/aje/kwf113 12397006

[44] Govindaraju DR, Cupples LA, Kannel WB, O’Donnell CJ, Atwood LD, D’Agostino RB Sr, Fox CS, Larson M, Levy D, Murabito J, Vasan RS, Splansky GL, Wolf PA, Benjamin EJ. Genetics of the Framingham Heart Study population. Adv Genet. 2008; 62:33–65. 10.1016/S0065-2660(08)00602-0 19010253 PMC3014216

[45] Splansky GL, Corey D, Yang Q, Atwood LD, Cupples LA, Benjamin EJ, D’Agostino RB Sr, Fox CS, Larson MG, Murabito JM, O’Donnell CJ, Vasan RS, Wolf PA, Levy D. The Third Generation Cohort of the National Heart, Lung, and Blood Institute’s Framingham Heart Study: design, recruitment, and initial examination. Am J Epidemiol. 2007; 165:1328–35. 10.1093/aje/kwm021 17372189

[46] Cupples LA, Heard-Costa N, Lee M, Atwood LD, and Framingham Heart Study Investigators. Genetics Analysis Workshop 16 Problem 2: the Framingham Heart Study data. BMC Proc. 2009 (Suppl 7); 3:S3. 10.1186/1753-6561-3-s7-s3 20018020 PMC2795927

[47] Juster FT, Suzman R. An overview of the health and retirement study. J Hum Resour. 1995; 30:S7–56. 10.2307/146277

[48] Howie BN, Donnelly P, Marchini J. A flexible and accurate genotype imputation method for the next generation of genome-wide association studies. PLoS Genet. 2009; 5:e1000529. 10.1371/journal.pgen.1000529 19543373 PMC2689936

[49] Fisher RA. 1970. Statistical methods for research workers. (Edinburgh: Oliver and Boyd).

[50] Shi G, Rice TK, Gu CC, Rao DC. Application of three-level linear mixed-effects model incorporating gene-age interactions for association analysis of longitudinal family data. BMC Proc. 2009 (Suppl 7); 3:S89. 10.1186/1753-6561-3-s7-s89 20018085 PMC2795992

[51] Bates D, Machler M, Bolker BM, Walker SC. Fitting Linear Mixed-Effects Models Using lme4. J Stat Softw. 2015; 67:1–48. 10.18637/jss.v067.i01

[52] Prentice R, Breslow N. Retrospective studies and failure time models. Biometrika. 1978; 65:153–58. 10.1093/biomet/65.1.153

[53] Ikram MA, Seshadri S, Bis JC, Fornage M, DeStefano AL, Aulchenko YS, Debette S, Lumley T, Folsom AR, van den Herik EG, Bos MJ, Beiser A, Cushman M, et al Genomewide association studies of stroke. N Engl J Med. 2009; 360:1718–28. 10.1056/NEJMoa0900094 19369658 PMC2768348

[54] Yashin AI, Wu D, Arbeev KG, Arbeeva LS, Akushevich I, Kulminski A, Culminskaya I, Stallard E, Ukraintseva SV. Genetic Structures of Population Cohorts Change with Increasing Age: Implications for Genetic Analyses of Human aging and Life Span. Ann Gerontol Geriatr Res. 2014; 1. 25893220 PMC4398390

[55] Willer CJ, Li Y, Abecasis GR. METAL: fast and efficient meta-analysis of genomewide association scans. Bioinformatics. 2010; 26:2190–91. 10.1093/bioinformatics/btq340 20616382 PMC2922887

[56] Begum F, Ghosh D, Tseng GC, Feingold E. Comprehensive literature review and statistical considerations for GWAS meta-analysis. Nucleic Acids Res. 2012; 40:3777–84. 10.1093/nar/gkr1255 22241776 PMC3351172

[57] Fortney K, Dobriban E, Garagnani P, Pirazzini C, Monti D, Mari D, Atzmon G, Barzilai N, Franceschi C, Owen AB, Kim SK. Genome-Wide Scan Informed by Age-Related Disease Identifies Loci for Exceptional Human Longevity. PLoS Genet. 2015; 11:e1005728. 10.1371/journal.pgen.1005728 26677855 PMC4683064

[58] Xu X, Tian L, Wei LJ. Combining dependent tests for linkage or association across multiple phenotypic traits. Biostatistics. 2003; 4:223–29. 10.1093/biostatistics/4.2.223 12925518

[59] Bolormaa S, Pryce JE, Reverter A, Zhang Y, Barendse W, Kemper K, Tier B, Savin K, Hayes BJ, Goddard ME. A multi-trait, meta-analysis for detecting pleiotropic polymorphisms for stature, fatness and reproduction in beef cattle. PLoS Genet. 2014; 10:e1004198. 10.1371/journal.pgen.1004198 24675618 PMC3967938

[60] Zhu X, Feng T, Tayo BO, Liang J, Young JH, Franceschini N, Smith JA, Yanek LR, Sun YV, Edwards TL, Chen W, Nalls M, Fox E, et al and COGENT BP Consortium. Meta-analysis of correlated traits via summary statistics from GWASs with an application in hypertension. Am J Hum Genet. 2015; 96:21–36. 10.1016/j.ajhg.2014.11.011 25500260 PMC4289691

[61] Zhu X, Feng T, Tayo BO, Liang J, Young JH, Franceschini N, Smith JA, Yanek LR, Sun YV, Edwards TL, Chen W, Nalls M, Fox E, et al and COGENT BP Consortium. Meta-analysis of correlated traits via summary statistics from GWASs with an application in hypertension. Am J Hum Genet. 2015; 96:21–36. 10.1016/j.ajhg.2014.11.011 25500260 PMC4289691

[62] Leslie R, O’Donnell CJ, Johnson AD. GRASP: analysis of genotype-phenotype results from 1390 genome-wide association studies and corresponding open access database. Bioinformatics. 2014; 30:i185–94. 10.1093/bioinformatics/btu273 24931982 PMC4072913

