# Supplementary material for: Strong impact of natural-selection–free heterogeneity in genetics of age-related phenotypes
Source: Aging (Albany NY). 2018 Mar 29;10(3):492–514. doi: 10.18632/aging.101407 (PMC5892700; doi:10.18632/aging.101407)
Supplement: Supplementary File [file aging-10-101407-s001.pdf]

SUPPLEMENTARY MATERIAL

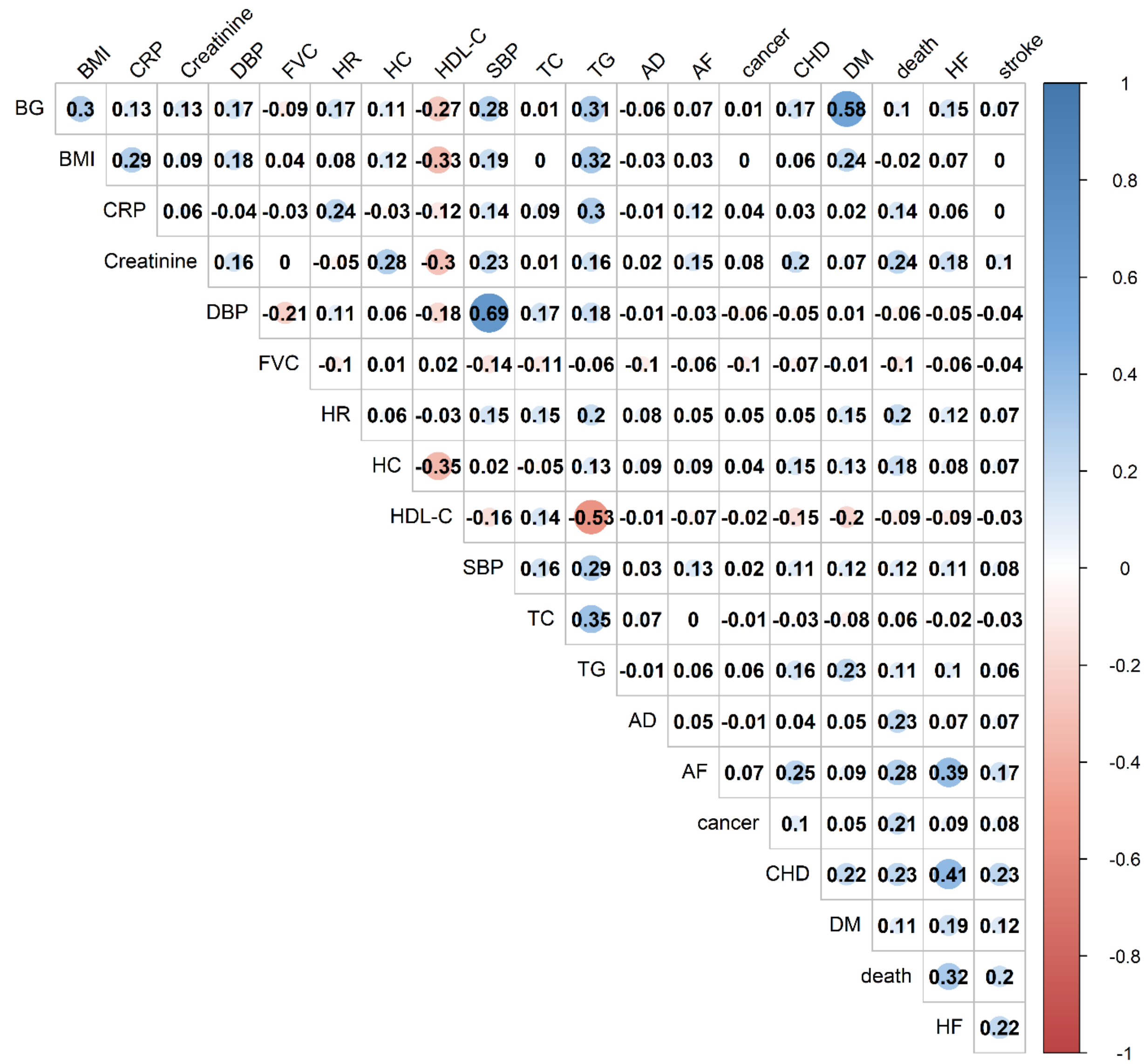

Supplementary Figure 1. Pairwise correlation between phenotypes selected for the analysis. Correlation coefficients are from correlation matrix $\mathbf{\Sigma}_m^P$ evaluated using phenotype measurements in the combined data from all cohorts (see Online Methods). Quantitative markers: blood glucose (BG); body mass index (BMI); C-reactive protein (CRP); creatinine; diastolic blood pressure (DBP); forced vital capacity (FVC); heart rate (HR); hematocrit (HC); high-density lipoprotein cholesterol (HDL-C); systolic blood pressure (SBP); total cholesterol (TC); and triglycerides (TG). Risk outcomes: Alzheimer's disease and related dementias (AD), atrial fibrillation (AF); cancer; coronary heart disease (CHD); diabetes mellitus (DM); death; heart failure (HF); and stroke.

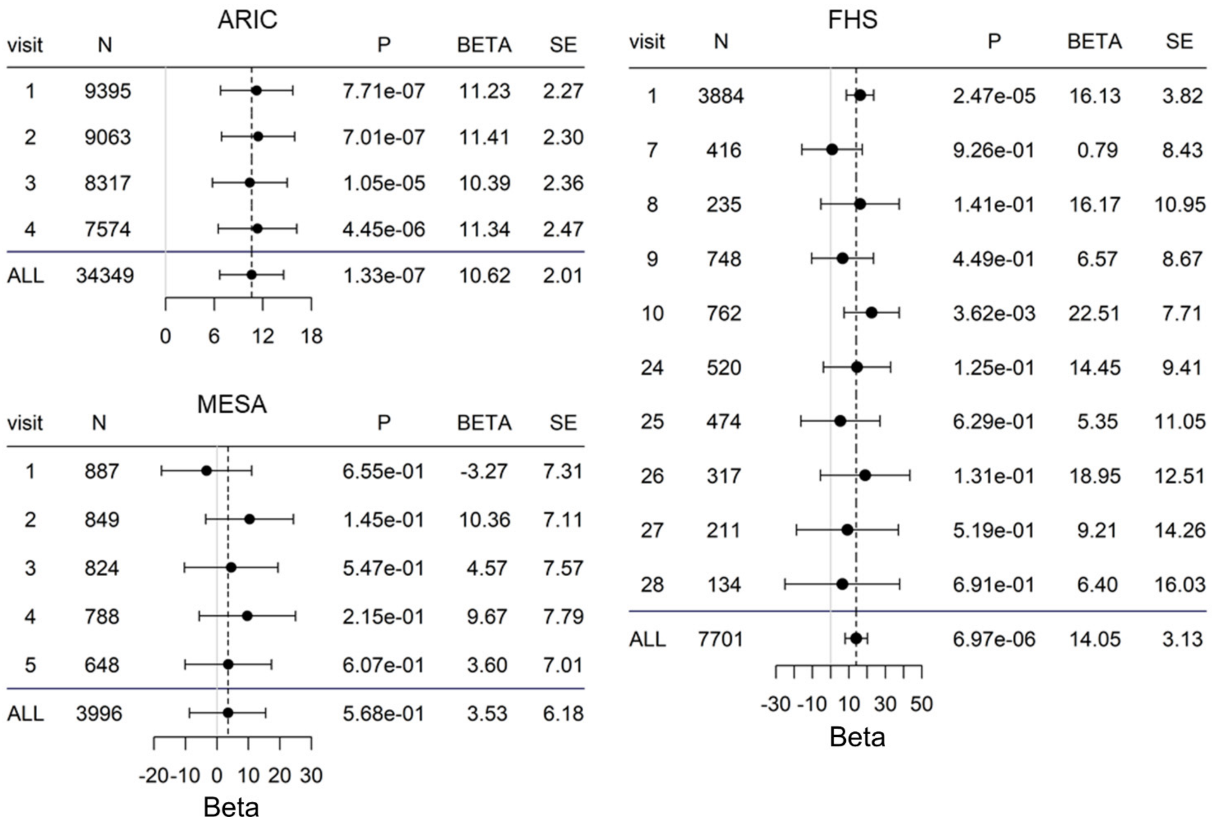

Supplementary Figure 2. Forest plots illustrating benefits of longitudinal information for rs2155216. Cohorts: Atherosclerosis Risk in Communities Study (ARIC); Framingham Heart Study (FHS), and the Multi-Ethnic Study of Atherosclerosis (MESA). N indicates the sample size at a given examination (visit) and person-observations. “All” denotes the estimates evaluated leveraging longitudinal information. SE denotes standard error. Bars show 95% confidence intervals. The *x*-axis shows effects sizes beta.

Supplementary Table 1. Basic characteristics of cohorts included in the analyses and available sample sizes.

Variable	ARIC	CHS	FHS	MESA	HRS
Sample size	9,612	3,182	8,628	2,527	9,482
Age (SD), years	54.3 (5.7)	72.4 (5.4)	37.8 (9.3)	64.3 (10.2)	58.2 (9.1)
Birth dates, range	1921-1944	1885-1925	1885-1980	1917-1957	1905-1974
Gender, female (%)	5,095 (53.0)	1,841 (60.4)	4,659 (54.0)	1,320 (52.3)	5,489 (57.9)
Quantitative markers: number of visits (number of person-visits available for the analyses)					
BG [mg/dl]	4 (35,090)	3 (7,515)	17 (40,065)	5 (11,180)	1 (7,404)
BMI [kg/m^2]	4 (35,055)	3 (7,570)	19 (46,187)	5 (11,200)	1 (9,419)
CRP [mg/l]	1 (523)	2 (5,564)	3 (18,699)	2 (2,716)	1 (8,087)
Creatinine [mg/dl]	3 (26,603)	3 (7,515)	6 (23,210)	4 (8,816)	N/A
DBP [mmHg]	4 (35,109)	7 (21,872)	28 (54,740)	5 (11,234)	2 (18,806)
FVC [liter]	2 (18,668)	3 (6,915)	4 (4,813)	1 (1,324)	N/A
HC [%]	3 (26,529)	2 (5,934)	14 (15,070)	1 (396)	N/A
HDL-C [mg/dl]	4 (35,033)	2 (5,611)	8 (35,548)	5 (11,166)	1 (5,973)
HR [beat/min]	4 (34,966)	7 (21,961)	25 (47,932)	2 (4,291)	N/A
SBP [mmHg]	4 (35,109)	7 (21,922)	28 (54,751)	5 (11,234)	2 (18,806)
TC [mg/dl]	4 (35,065)	6 (16,258)	18 (46,287)	5 (11,173)	1 (7,067)
TG [mg/dl]	4 (35,082)	2 (5,617)	4 (7,728)	5 (11,177)	N/A

Risk outcomes: number of cases available for the analyses					
Follow up through	2005	2003	2014	2012	2013
AD	N/A	185	437	N/A	261
AF	837	631	957	137	N/A
Cancer	1,298*	631	1,366	N/A	1,640
CHD	1,497	952	1,140	170	2,644
DM	862	406	709	382	1,956
Death	1,430	1,345	1,784	176	1,239
HF	823	676	596	72	1,360
Stroke	398	300	462	57	1,232

* All cancer sites combined; otherwise, all cancer sites except skin.

Cohort: Atherosclerosis Risk in Communities Study (ARIC); Cardiovascular Health Study (CHS); Framingham Heart Study (FHS), the Multi-Ethnic Study of Atherosclerosis (MESA), and Health and Retirement Study (HRS).

Quantitative markers: blood glucose (BG); body mass index (BMI); C-reactive protein (CRP); creatinine; diastolic blood pressure (DBP); forced vital capacity (FVC); heart rate (HR); hematocrit (HC); high-density lipoprotein cholesterol (HDL-C); systolic blood pressure (SBP); total cholesterol (TC); and triglycerides (TG).

Risk outcomes: Alzheimer's disease and related dementias (AD), atrial fibrillation (AF); cancer; coronary heart disease (CHD); diabetes mellitus (DM); death; heart failure (HF); and stroke.

N/A identifies phenotypes not available in a given cohort.

Age is given at baseline; standard deviation (SD).

Supplementary Table 2. Linkage disequilibrium (LD, r2) for all non-proxy SNPs reported in the paper, which are within ±1Mb flanking region for the index SNP.

SNP_A	Chr_A	BP_A	SNP_B	Chr_B	BP_B	LD, r^2
rs17367504	1	11802721	rs17376328	1	11816605	0.228
rs7549339	1	31249319	rs16834550	1	31530916	0.028
rs780094	2	27518370	rs780092	2	27520287	0.138
rs755503	2	134386883	rs503562	2	134502500	0.091
rs755503	2	134386883	rs666614	2	134532882	0.070
rs755503	2	134386883	rs6745983	2	134532882	0.068
rs755503	2	134386883	rs6430585	2	134673139	0.037
rs503562	2	134502500	rs666614	2	134673139	0.219
rs503562	2	134502500	rs6745983	2	134673139	0.130
rs503562	2	134502500	rs6430585	2	135749357	0.040
rs666614	2	134532882	rs6745983	2	135749357	0.399
rs666614	2	134532882	rs6430585	2	135749357	0.115
rs6745983	2	134673139	rs6430585	2	135749357	0.125
rs2844720	6	30507940	rs6936620	6	33016674	0.007
rs2410616	8	19971168	rs17482753	8	19975135	0.019
rs2410616	8	19971168	rs17410962	8	19990569	0.020
rs2410616	8	19971168	rs17489268	8	19990569	0.465
rs17482753	8	19975135	rs17410962	8	19994534	0.639
rs17482753	8	19975135	rs17489268	8	19994534	0.251
rs17410962	8	19990569	rs17489268	8	19994534	0.190
rs2155216	11	116661022	rs6589567	11	116799960	0.101
rs2155216	11	116661022	rs7115242	11	117037567	0.043
rs6589567	11	116799960	rs7115242	11	117037567	0.232
rs9315885	13	42068674	rs670676	13	42127603	0.248
rs261332	15	58435126	rs261336	15	58450219	0.509

BP = base pairs, Chr = chromosome.

Linkage disequilibrium (LD) was evaluated with cut off r^2=0.01 in the Framingham Heart Study.

ADDITIONAL SUPPLEMENTARY MATERIAL

Please browse the Full Text version to see the links to Supplementary Tables of this manuscript.

Supplementary Table 3. SNPs attained genome-wide significance ($p<5E-8$) in univariate meta-analysis of individual phenotypes.

Supplementary Table 4. Replicated SNPs attained genome-wide significance in pleiotropic meta-analysis. (**A**) Replicated SNPs attained genome-wide significance in pleiotropic meta-analysis. (**B**) Effect sizes and p-values from the univariate meta-analysis of the associations of SNPs from Supplementary Table 4A with 20 phenotypes in pathway 1a using fixed-effects meta-test. (**C**) P-values from the univariate meta-analysis of the associations of SNPs from Supplementary Table 4A with 20 phenotypes in pathway 1a using Fisher's test. (**D**) The results of pleiotropic meta-analysis for SNPs from Supplementary Table 4A in each phenotypic domain.

Supplementary Table 5. Results of univariate meta-analysis of the associations of SNPs from Table 2 with 20 phenotypes in pathway 1a. (**A**) Effect sizes and p-values from the univariate meta-analysis of the associations of SNPs from Table 2 with 20 phenotypes in pathway 1a using fixed-effects meta-test. (**B**) P-values from the univariate meta-analysis of the associations of SNPs from Table 2 with 20 phenotypes in pathway 1a using Fisher's test.

Supplementary Table 6. The results of pleiotropic meta-analysis for SNPs from Table 2 in each phenotypic domain.

Supplementary Table 7. The results of pleiotropic meta-analyses for SNPs reported in Table 2 for pathway 2 in each cohort separately (pathway 2a) and after Fisher meta-analysis across cohorts (pathway 2b).

Supplementary Table 8. Pleiotropic meta-statistics for the antagonistic heterogeneity reported in Table 2 for pathway 2 in each cohort separately and combined using three meta-tests, FpFc, OpFc, and ObFc in pathway 2b (Figure 1).

Supplementary Table 9. Ten nominally significant gene ontology (GO) terms (Fisher’s exact test) for biological processes enriched in DAVID.

Supplementary Table 10. Top 18 nominally significant (Fisher's exact test) ingenuity canonical pathways.
